# Tonabersat enhances temozolomide‐mediated cytotoxicity in glioblastoma by disrupting intercellular connectivity through connexin 43 inhibition

**DOI:** 10.1002/1878-0261.13786

**Published:** 2024-12-16

**Authors:** Elena N. C. Schmidt, Bernd O. Evert, Barbara E. F. Pregler, Ahmad Melhem, Meng‐Chun Hsieh, Markus Raspe, Hannah Strobel, Julian Roos, Torsten Pietsch, Patrick Schuss, Pamela Fischer‐Posovszky, Mike‐Andrew Westhoff, Michael Hölzel, Ulrich Herrlinger, Hartmut Vatter, Andreas Waha, Matthias Schneider, Anna‐Laura Potthoff

**Affiliations:** ^1^ Department of Neurosurgery University Hospital Bonn Germany; ^2^ Brain Tumor Translational Research Group University Hospital Bonn Germany; ^3^ Department of Neurology University Hospital Bonn Germany; ^4^ Department of Pediatrics and Adolescent Medicine University Medical Center Ulm Germany; ^5^ Department of Neuropathology University Hospital Bonn Germany; ^6^ German Center for Child and Adolescent Health (DZKJ), partner site Ulm Germany; ^7^ Institute of Experimental Oncology University Hospital Bonn Germany; ^8^ Department of Neurooncology, Center for Neurology and Center of Integrated Oncology ABCD University Hospital Bonn Germany; ^9^ Present address: Department of Neurosurgery BG Klinikum Unfallkrankenhaus Berlin BG Germany

**Keywords:** cell death, connexin 43, glioblastoma, tonabersat, tumor microtubes, tumor networks

## Abstract

Glioblastoma cells rely on connexin 43 (Cx43)‐based gap junctions (GJs) for intercellular communication, enabling them to integrate into a widely branched malignant network. Although there are promising prospects for new targeted therapies, the lack of clinically feasible GJ inhibitors has impeded their adoption in clinical practice. In the present study, we investigated tonabersat (TO), a blood–brain‐barrier‐penetrating drug with GJ‐inhibitory properties, in regard to its potential to disassemble intercellular connectivity in glioblastoma networks. Fluorescence‐guided measurements of calcein cell‐to‐cell transfer were used to study functional intercellular connectivity. Specific DNA fragmentation rates of propidium iodide‐stained nuclei were measured as a surrogate readout for cell death using flow cytometry. CRISPR/Cas9‐mediated gene editing of Cx43 served as a validation tool of cellular effects related to Cx43 GJ inhibition. 3′ mRNA sequencing was performed for molecular downstream analysis. We found that TO reduced intercellular GJ‐mediated cytosolic traffic and yielded a significant reduction of tumor microtube (TM) length. TO‐mediated inhibition of cellular tumor networks was accompanied by a synergistic effect for temozolomide‐induced cell death. CRISPR/Cas9 *Cx43*‐knockout revealed similar results, indicating that TO‐mediated inhibitory effects rely on the inhibition of Cx43‐based GJs. Gene set enrichment analyses found that GJ‐mediated synergistic cytotoxic effects were linked to a significant upregulation of cell death signaling pathways. In conclusion, TO disrupts TM‐based network connectivity via GJ inhibition and renders glioblastoma cells more susceptible to cytotoxic therapy. Given its previous use in clinical trials for migraine therapy, TO might harbor the potential of bridging the idea of a GJ‐targeted therapeutic approach from bench to bedside.

## Graphical abstract

Tonabersat (TO) inhibits Cx43‐based gap junctions (GJs) in glioblastoma, leading to both functional and morphological breakdown of malignant cell‐to‐cell connectivity. This increases sensitivity to temozolomide (TMZ)‐induced cell death, reduces tumor cell proliferation, and enhances TMZ‐activated molecular pathways that drive growth arrest and apoptosis. TO's potential as a clinically feasible drug holds promise for advancing new therapeutic approaches in glioblastoma treatment.

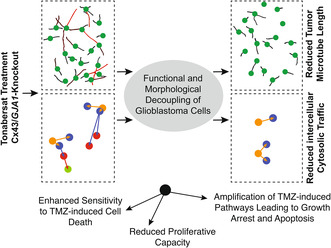

**Abbreviations**
BrdU: bromodeoxyuridine
CRISPR: clustered regularly interspaced short palindromic repeats
Cx43: Connexin43
DEG: differentially expressed genes
DMSO: dimethyl sulfoxide
DPBS: Dulbecco's phosphate‐buffered saline
dsDNA: double‐strand DNA
FC: fold change
FCS: fetal calf serum
GBM: glioblastoma
*GJA1*
: gene encoding for Cx43, synonym for protein connexin 43
GJs: gap junctions
gRNA: guide RNA
GSEA: gene set enrichment analysis
hrs: hours
IC50: half maximal inhibitory concentration
IQR: interquartile range
KO: knockout
log2FC: log 2 fold change
MFA: meclofenamate
Mins: minutes
mRNA: messenger RNA
MTT: 3‐(4,5‐Dimethylthiazol‐2‐yl)‐2,5‐diphenyltetrazoliumbromid
MULE: multiple lentiviral expression
*P*‐adj: adjusted *P*‐value
PCA: principial component analysis
PI: propidiumiodide
PVDF: polyvinylidenfluoride
RMA: robust multi‐array average
RT: room temperature
SDS: sodium dodecyl sulfate
sgRNA: single guide RNA
TBS(T): tris‐buffered saline (with 0.1% Tween 20)
TCGA: The Cancer Gene Atlas
TM: tumor microtubes
TMZ: temozolomide
TNT: tunneling nanotubes
TO: tonabersat
VST: variance stabilizing transformation
WT: wild‐type

## 1 Introduction

In recent years, the discovery of functional and communicative cellular tumor networks has fundamentally changed our understanding of brain tumor biology [1]. In glioblastoma, these networks are based on the formation of tumor microtubes (TMs) as ultra‐long and thin membrane protrusions [2], which connect tumor cells both with each other and with their cellular microenvironment [1]. The interconnecting points between TMs are supported by intercellular gap junctions (GJs) composed of two connexin 43 (Cx43, also known as gap junction alpha‐1 protein, GJA1) hexamers [1]. The formation of tumor networks through GJ‐coupled TMs has been shown to contribute to tumor growth, brain microinvasion, and resistance to anticancer therapies [3, 4, 5] entailing promise for novel and expanded multimodal therapeutic regimens [6, 7, 8, 9]. By enabling direct intercellular communication routes in the malignant cellular tumor network, GJs facilitate the transfer of pro‐tumorigenic signals and promote the development of a microinvasive and resistant phenotype [10, 11]. Therefore, with the current knowledge that glioblastomas are arranged as a functional and communicative cellular network based on TM interconnected via GJs, these GJs have gained growing interest as a potential therapeutic target for the treatment of glioblastoma patients.

However, the lack of clinically feasible GJ inhibitors has hindered progress in this field. Tonabersat (TO), a benzopyran derivative with GJ‐inhibitory properties [12, 13], might constitute a promising candidate for targeting intercellular connectivity in malignant brain tumors. A major advantage of TO is its ability to cross the blood–brain barrier [13, 14], making it a potential treatment option for glioblastoma. TO was evaluated in a preclinical rat model for glioblastoma, demonstrating a survival advantage when combined with the standard treatment regimen of radiation and temozolomide (TMZ) [15, 16]. However, no functional analysis was conducted in this study. Furthermore, TO has been extensively studied in clinical trials for migraine, where it has been shown to reduce the frequency and severity of migraine attacks, possibly by inhibiting GJ coupling between neurons and glial cells [17, 18]. This suggests that TO may also be effective in inhibiting GJ coupling between glioblastoma cells.

In the present study, we show that TO inhibits intercellular GJ‐mediated cytosolic exchange in preformed glioblastoma networks culminating in a synergistic effect for cytotoxic therapy. Interestingly, by knocking out endogenous Cx43 gene expression in glioblastoma cells using CRISPR/Cas9‐mediated genome editing, we observed similar responses in Cx43‐knockout cells treated with TMZ alone as in WT cells treated with TO and TMZ combined. These findings indicate that TO‐mediated sensitizing effects are attributed to the inhibition of Cx43‐based GJs.

Given its assessable side‐effect profile known from clinical trials in the field of migraine therapy, these findings suggest TO as a promising and clinically feasible drug to disrupt the malignant network connectivity in glioblastoma.

## 2 Materials and methods

### 2.1 Cell culture

Human primary glioblastoma cell populations were isolated by mechanical dissociation from surgically obtained fresh tumor tissue as part of routine medical procedures at the University Hospital Ulm between 2010 and 2013, as previously described [19]. The three glioblastoma cell populations used for further experiments (G35, G71, and G106) were prepared from tumors classified as glioblastoma, CNS WHO grade 4, *IDH*‐WT according to the current WHO Classification of Tumors of the CNS 5^th^ edition published in 2021 [20]. The research methods employed in this study adhered to the ethical standards outlined in the Declaration of Helsinki. The processing previously extracted brain tumor tissue from routine medical procedures for research purposes has been approved by the ethics committee of the University Hospital Ulm (No. 162/10). Written informed consent was obtained from all patients after they have been properly instructed.

Glioblastoma cells were cultured in DMEM/F12 medium (Gibco, Waltham, MA, USA) supplemented with EGF (0.2 μg·mL^−1^) (Biomol, Hamburg, Germany), FGF (0.1 μg·mL^−1^) (Miltenyi Biotec GmbH, Bergisch Gladbach, Germany), B27 supplement without Vitamin A (1%) (Gibco), the antifungal agent amphotericin B (2%) (Gibco) and penicillin/streptomycin (1%) (Pan Biotech GmbH, Aidenbach, Germany). The suspension cells were induced to differentiate by transferring them into ventilated cell culture flasks (Corning™ Falcon™, New York, NY, USA) containing DMEM medium (Gibco) supplemented with Fetal Calf Serum (FCS) (10%) (Gibco) and penicillin/streptomycin (1%). In this way, the cells adhered and were used for the described experiments for a maximum of 12 cell passages [19]. Both suspension and adherent cells were cultured in a humidified incubator at 37 °C containing 5% CO_2_.

### 2.2 Characterization of glioblastoma cell populations

All cell populations used were characterized for their *MGMT* promoter methylation status and the presence of an *IDH1* and *IDH2* mutation by pyrosequencing. Genomic DNA was extracted using the QIAamp DNA Mini Kit (Qiagen, Hilden, Germany) according to the instructions of the manufacturer. The analysis of the somatic mutations at codon R132 (*IDH1*) and R172 (*IDH2*) was performed by pyrosequencing as previously described [21]. For the analysis of the *MGMT* promotor methylation, DNA was subjected to bisulfite conversion and analyzed by pyrosequencing as previously described [22]. Sequencing was performed on a Pyromark Q24 instrument (Qiagen) [3].

### 2.3 Protein immunoblotting

Soluble protein for western blotting was harvested from cells lysed for 30 min on ice in lysis buffer containing 1 m NaCl, 1 m Tris–Hcl pH 7.5, 20% Triton, 1 m MgCl, Halt™ (Protease Inhibitor, Thermo Fisher Scientific, Waltham, MA, USA, 78429, dilution 1 : 100) and Benzonase (1 : 2000). The lysates were then centrifugated at full speed for 10 min at 4 °C. The protein concentration was determined using the BCA Assay (Thermo Fisher Scientific, 23227). For electrophoresis, 50 μg of protein from each sample was mixed with Laemmli loading buffer, boiled for 5 min at 95 °C to denature the proteins, and then separated on a 10% SDS/PAGE gel. The separated proteins were transferred to PVDF membranes. Immunodetection involved blocking the membranes for 1 h at RT in a blocking solution containing 5% [bovine serum albumin (BSA) in TBST (Tris‐buffered saline with 0.1% Tween 20)]. Following blocking, the membranes were incubated overnight at 4 °C with primary antibodies, including anti‐Cx43 (Sigma, St. Louis, MO, USA, C‐6219, diluted 1 : 1000) and anti‐β‐actin (Sigma, A5441, diluted 1 : 10 000) as loading controls. After several washes in TBST, the membranes were incubated for 1 h at room temperature (RT) either with the appropriate secondary antibody conjugated with horseradish peroxidase (polyclonal goat anti‐mouse‐HRP, Agilent Dako, Santa Clara, CA, USA, #P0447, anti‐rabbit‐HRP Cell Signaling, Danvers, MA, USA, #7074, both diluted 1 : 5000) or with a fluorophor‐conjugated secondary antibody [Thermo Fisher Scientific, Alexa 647 (A21236) and Alexa 488 (A11034), both diluted 1 : 10 000] diluted in TBST. The membranes were then washed 3 times for 5 min in TBST and once again with TBS. The protein bands were visualized and analyzed either using the chemiluminescent substrate added to the membranes visualized with the ChemoCam Imager (Intas) for G35, G71, and G106 WT cells as well as G35 KO clones #6 and #11 as previously reported [3]. For the verification of G71 KO clones #2.3 and #2.7, proteins were separated using a 12% Tris‐Glycine SDS/PAGE gel. Cx43 was detected with the same primary antibody previously utilized, GAPDH served as the loading control and was detected using a monoclonal antibody (HyTest Ltd, Turku, Finland, 5G4Mab6C5). The secondary antibodies applied were StarBright Blue 700 Goat Anti‐Mouse IgG (Bio‐Rad Laboratories, Munich, Germany, 12004158) and StarBright Blue 520 Goat Anti‐Rabbit IgG (Bio‐Rad Laboratories, 12005869). The membranes were visualized using the ChemiDoc™ Device (Bio‐Rad Laboratories).

### 2.4 Immunofluorescence

For immunofluorescence imaging, differentiated glioblastoma cells were seeded in 6‐well plates (Sarstedt, Nümbrecht, Germany) at a density of 4 × 10^5^ cells per well on glass coverslips (24 × 60 mm) (Sigma). The adherent cells were fixed after 3 days with 16% paraformaldehyde solution (Sigma) at 37 °C for 15 min followed by permeabilization with 0.1% Triton X‐100 (Sigma) and washing once with DPBS (Gibco). To prevent non‐specific antibody binding, blocking was performed with a 5% BSA solution (Carl Roth, Karlsruhe, Germany). The primary antibody, diluted in 2% BSA (Carl Roth), rabbit polyclonal anti‐connexin‐43 (1 : 500) (Sigma, C6219‐25UL) and mouse monoclonal anti‐GFAP (1 : 200) (Sigma, G3893‐100UL) were added to the coverslips and incubated for 1 h protected from light at RT. Subsequently, coverslips were washed three times with DPBS and each was incubated with 50 μL of the secondary antibody goat anti‐rabbit Alexa Fluor 488 (1 : 500) (Sigma, A11034) and goat anti‐mouse Alexa Fluor 647 (1 : 500) (Sigma, A21236) diluted in 2% BSA, for 45 min protected from light at RT. Finally, the nuclei were stained. Therefore, cells were incubated with DAPI (1 μg·mL^−1^) (Southern Biotech, Birmingham, AL, USA) diluted in DPBS for 5 min protected from light at RT. After washing twice in DPBS and once in H_2_O, the coverslips were finally mounted on slides with Prolong Gold (Gibco).

Confocal images were acquired on the spinning disc microscope (VisiScope CSU‐W1, Nikon, Europe), with the 10×, 20× and 40× objective. The intensity of the Cx43 channel was evaluated semiquantitative from 20× images by imagej, (U.S. National Institutes of Health, Bethesda, MD, USA).

### 2.5 Analysis of publicly available datasets

Using the GlioVis data portal [23], the RNA sequencing datasets HG‐U133A and Agilent‐4502A from TCGA [24], as well as datasets from the Rembrandt (GSE108474) [25] and Gravendeel [26] databases, were analyzed for mRNA expression levels of *GJA1* in glioblastoma and non‐tumor samples. For the TCGA datasets, the subgroup of primary glioblastomas, *IDH*‐WT, and *MGMT*‐hypermethylated were specifically analyzed. No further differentiation between glioblastoma samples was performed for the Rembrandt and Gravendeel databases [25, 26]. Adjusted *P*‐values were calculated using Tukey's Honest Significant Difference test. Boxplots were directly downloaded from the on‐website analysis. Correlation analysis of *GJA1* with other genes was performed using the GlioVis data portal as well.

For the analysis of mRNA expression levels encoding for various connexins, the Affymetrix expression array HG‐U133A was utilized. Raw .CEL files were downloaded from their respective sources, and the analyses were conducted in r. The ‘affy’ package was employed for robust multi‐array average (RMA) normalization, followed by quantile normalization [27]. For genes with multiple probe sets, the median value of all probes was selected. The *P*‐value for *GJA1* expression compared to other connexin genes was calculated using the one‐way ANOVA test. To further confirm the differences in expression levels, Dunnett's Multiple Comparison Test was employed (*P*‐adj value).

Additionally, we analyzed the Agilent‐4502A dataset to investigate the correlation between *GJA1* expression and several genes that were significantly upregulated following treatment with TMZ and TO. We employed the Pearson correlation method, as detailed on the respective website, calculating both the *P*‐value and the correlation coefficient (*r*).

### 2.6 Inhibitors and drugs

Temozolomide (Sigma) and Tonabersat (Sigma) were both dissolved in dimethyl sulfoxide (DMSO) (Sigma) to obtain a 100 mm stock solution in the case of TMZ and a 50 mm stock solution in the case of TO. Both drugs were aliquoted and stored at −20 °C. In all experiments, concentrations of 50 μm TMZ, 50 μm TO in case of G35 and G71 and 100 μm TO in case of G106 cell population were used, unless indicated otherwise.

### 2.7 Cell viability assay (MTT)

Cell viability was monitored by MTT assay (Sigma, G3581) by measuring the reduction of a tetrazolium salt (3‐(4,5‐dimethylthiazol‐2yl)‐2,5‐diphenyltetrazolium bromide) to formazan as described previously [28]. Primary cells were seeded in flat bottom 96‐well plates (Sarstedt) at a density of 1.5 × 10^3^ cells per well and treated with the specified concentrations of TMZ and TO. Cell viability was determined 72 and 144 h after treatment by adding 20 μL MTT solution (5 mg·mL^−1^ in DPBS) (Sigma) to each well. After a 3‐h incubation at 37 °C, the supernatant was discarded and cells were lysed by adding 200 μL DMSO. The reduction of MTT by viable cells was quantified at 560 nm and a reference wavelength of 620 nm using a μQuant microplate spectrophotometer (BioTek Instruments, USA) [28]. All experiments were performed as a biological and technical triplicate.

### 2.8 Calcein dye transfer

The fluorescent dye calcein AM (Thermo Fisher Scientific) was used to study cell‐to‐cell communication via intercellular GJs through live cell imaging, following a previously protocol [29]. For time course analysis, 1 × 10^5^ unlabeled acceptor cells were seeded in 35 mm tissue culture dishes and after 48 h pre‐incubation cells were treated with TO and TMZ individually or in combination. Donor cells (Calcein stained cells) were prepared by staining with calcein (5 μm) for 20 min and then added to the dishes in a 1:4 ratio. Fluorescence images were acquired every 20 min for a total of 210 min using a Lionheart FX automated microscope (BioTek/Agilent, Santa Clara, CA, USA). Quantitative analysis of the fluorescence images was performed to determine the number of receiver cells (acceptor cells that took up the calcein dye) using a customized Cell Profiler pipeline [30]. The cellular distribution of calcein was calculated by dividing the number of receiver cells by the number of donor cells. Visualization of dye spreading was performed with a customized R pipeline.

### 2.9 Analysis of tumor microtubes

To quantify the TM lengths and number of individual cells in cell culture, fluorescence images were acquired using the Lionheart FX microscope (BioTek/Agilent). Cells were seeded at a density of 3 × 10^4^ cells per well into a 96‐well plate. Treatment was started 24 h afterwards and measurement of TM length and number was performed after 48 and 96 h of treatment. Obtained images were then analyzed using the Neuroanatomy SNT plugin within imagej [31] to measure both the lengths and number of TMs. Further processing of obtained data tables was performed using r.

### 2.10 Flow Cytometric analysis of cell death

DNA fragmentation was used as readout for cell death and assessed by flow cytometric (FACS Canto II, Becton Dickinson, Heidelberg, Germany) measurement of propidium iodide‐stained nuclei as previously described [19]. Briefly, cells were seeded in 12‐well plates (Sarstedt) with a density of 8 × 10^3^ cells per well. After 24‐h incubation, the medium was replaced to contain either TMZ (50 μm), TO (50/100 μm) or a combination of both. After 144 h, the cells were harvested, centrifuged [5 min, 1300 rpm (or approximately 327 ***g***), 4 °C], resuspended in Nicoletti buffer containing propidium iodide (50 μg·mL^−1^) (Acros Organics, Geel, Belgium) and 0.1% Triton‐X (Sigma) [32], and incubated at 4 °C for 1 h. Each experiment was performed in triplicates and independently repeated three times. The obtained data was analyzed using FlowJo software Version 10.4 (Ashland, Wilmington, DE, USA). Specific DNA fragmentation was calculated as follows: 100 × (experimental DNA fragmentation – spontaneous DNA fragmentation)/(100 − spontaneous DNA fragmentation) [19, 33]. Bliss Independence analysis was performed as previously described [4].

### 2.11 Cell proliferation assay (ELISA BrdU)

Cell proliferation was evaluated with Cell Proliferation Elisa, BrdU Kit (Roche, Grenzach‐Wyhlen, Germany). Primary glioblastoma cells (1.5 × 10^3^ cells per well) were seeded on a 96‐well plate and treated after 24 h with TMZ (50 μm) and TO (50/100 μm) either individually or in combination for 72 or 144 h. Subsequently, 10 μL per well BrdU Labeling Solution (100 μm) was added, and the cells were reincubated for additional 3 h at 37 °C. Afterwards, the culture medium was removed, the cells were fixed in FixDenat solution (200 μL per well) (30 min, RT). The solution of anti‐BrdU antibody coupled with horseradish peroxidase was subsequently added (100 μL per well) for 90 min at RT. Detection was performed using tetramethylbenzidine substrate (100 μL per well) according to the manufacturer's instructions. BrdU incorporation was quantitated colormetrically at 370 nm with a reference wavelength of 492 nm using a μQuant microplate spectrophotometer (Biotek Instruments).

### 2.12 Generation of *GJA1* /Cx43 knockout clones

#### 2.12.1 Knockout of G35 cells using the CRISPR/Cas9 method

Single guide RNA (sgRNA) sequences and potential CRISPR/Cas9 binding sites targeting exon 2 of the *GJA1* gene encoding for Cx43 near the transcription start site were selected from the UCSC genome browser (https://genome.ucsc.edu/) using the integrated CRISPOR tool (http://crispor.tefor.net). The sgRNA‐TS3 and sgRNA‐TS4 and their respective reverse complements (Table S1) were annealed, ligated and cloned into the Cas9 nuclease‐expressing GeneArt CRISPR Nuclease Vector according to manufacturer's protocol. To generate Cx43 KO cell clones, 1.5 × 10^6^ suspension cells from the primary G35 glioblastoma cell population were seeded on fibronectin (5 μg·cm^−2^) (Sigma) coated 10 cm tissue culture dishes. After 24 h, cells were cotransfected with 12.6 μg CRISPR‐Cas9‐gRNA‐Cx43 vector and 1.4 μg of the selection marker pTK‐hygro using RotiFect at a ratio of 1 : 5 (plasmids : RotiFect) in normal growth medium in a humidified 5% CO_2_ incubator. After 48 h, hygromycin B (0.025 mg·mL^−1^) was added to the transfected cells for the purpose of selecting single colonies. Single hygromycin‐resistant colonies were picked after approximately 2 weeks of cultivation and transferred into individual wells of fibronectin‐coated 24‐well plates. When picked colonies were confluent in 24‐well plates (approximately 5–7 days later), the colonies were split into two separate fibronectin‐coated 48‐well plates. One 48‐well plate was used for further expansion, while the other for analysis of Cx43 expression by immunoblotting as described above.

#### 2.12.2 Knockout of G71 cells using the multiple lentiviral expression system

CRISPR‐Cas9 constructs were generated using the MuLE system, employing the LR Gateway® cloning strategy [34] as previously described [35]. sgRNA duplexes targeting *GJA1*/Cx43, along with a non‐targeting control, were designed using the crispick online tool (Broad Institute, https://portals.broadinstitute.org/gppx/crispick/public) (Table S2). These duplexes were then inserted into the pMuLE ENTR U6 stuffer sgRNA scaffold L1‐R5 plasmid (Addgene, MA, USA, #1000000060, kindly provided by Ian Frew [34]) via ligation using Quick Ligase (New England Biolabs, Ipswich, MA, USA). Sanger sequencing confirmed the correct insertion of the sgRNA duplexes. The pMuLE ENTR U6‐sgRNA and pMuLE ENTR CMV‐hCas9 L5‐L2 plasmids were then recombined with a Sleeping Beauty transposon plasmid, ‘pMuSE eGFP‐P2A‐PuroR DEST’ [35], using LR Clonase II Plus (Thermo Fisher Scientific). G71 cells were cotransfected with the resulting pMuSE U6‐sgRNA+CMV‐hCas9 + RPBSA‐eGFP‐P2A‐PuroR plasmids and the Sleeping Beauty Transposase‐expressing pCMV(CAT)T7‐SB100 plasmid (Addgene, #34879, kindly provided by Zsuzsanna Izsvak) in a 19:1 mass ratio using the Neon Transfection System (Thermo Fisher Scientific) with three 10 ms pulses of 1300 V. Stable bulk cultures were selected after puromycin (1 μg·mL^−1^; Sigma) treatment, and single clones were expanded to achieve a complete knockout of Cx43.

### 2.13 Library preparation and RNA‐sequencing

For sequencing G35 cells were seeded at a density of 6 × 10^5^ in a 75 cm^2^ cell culture flask. Total RNA was isolated from G35 cells 48 h after treatment was started using the RNeasy mini kit (Qiagen). The quantity of RNA was assessed with a Nanodrop (Thermo Fisher Scientific) and RNA quality, indicated by an RNA Integrity Number (RIN) > 7, was verified by using a TapeStation System (Agilent). The library construction was performed using the QuantSeq FWD 3′‐mRNA‐Seq Kit (Lexogen, Vienna, Austria) according to the manufacturer's instructions (procedure executed by the NGS core facility, University Hospital Bonn) [36]. Each sample with 132–199 ng·μL^−1^ of total RNA was diluted in a volume of 5 μL containing the same concentration of total RNA. The RNA was hybridized to an oligo‐dT primer that carries an Illumina‐compatible sequence at its 5′ end, followed by reverse transcription. Once reverse transcription was completed, the RNA template was degraded and the synthesis of the second strand was initiated using a random primer equipped with an Illumina‐compatible linker at its 5′ end. Magnetic beads were used to purify the double‐stranded library, effectively removing all reaction components. The library was then amplified to include the full adapter sequences. Following PCR, another round of purification was conducted. Finally, high‐throughput sequencing was performed on the NovaSeq 6000 system, using single‐end 100 bp sequencing (Illumina, Inc., San Diego, CA, USA).

### 2.14 Data analysis (RNA sequencing)

For RNA sequencing analysis and visualization, the R/Bioconductor computing platform was used. Initial raw read counts, in a FASTQ file format, were aligned to the Hg38 human reference genome using the ‘rsubread’ package [37]. As suggested by Lexogen, the Rsubread align function was executed without trimming but allowing for mismatches in the initial cycles. For the final analysis, only those reads spanning a maximum length of 45 bases were included. The gene level summary was generated with unique mapping using the ‘featureCounts’ function. The aligned sequencing reads were then assigned to genomic features specified by an ENTREZ Gene ID (NCBI Gene database) [38]. Quality control steps such as variance stabilizing transformation (VST) and principal component analysis (PCA) have been applied. The differential gene expression analysis for drug responses (TMZ, TMZ + TO) was performed using the ‘deseq2’ package [39]. The results were filtered for a *P*‐adj < 0.05 and a log2FC > 0.5 in one approach and a log2FC > 2 in the other approach, and then displayed in a Volcano Plot (‘EnhancedVolcano’ function). In order to highlight the genes of certain interest, we generated boxplots using the ‘Limma’ package. Subsequently, we performed pairwise *t*‐tests to test for significant differences in mRNA expression levels between the different groups. In addition, GSEA was performed to identify classes of genes that are over‐ or under‐represented in the large set of genes to retrieve a functional profile to better understand the underlying processes after treatment with TMZ vs. TMZ + TO. GSEA was performed using the ‘ClusterProfiler’ package. GSEA plots were generated using the ‘gseaplot2’ function within the ‘enrichplot’ package in r. The link to access the processed data in form of a count matrix is available at https://www.ukbonn.de/neurochirurgie/forschung/onkologische‐forschung/.

### 2.15 Statistics and visualization

The statistical analyses were conducted using graphpad prism (version 9.5.1, GraphPad Software, LLC; Boston, MA, USA) and r (version R‐4.3.0, R Foundation, Vienna, Austria), while final figures were created using adobe illustrator (2023 version 27.7, Adobe Inc., San Jose, CA, USA). For comparisons involving more than two treatment conditions (but treatment as only variable), one‐way ANOVA with multiple testing was performed. The Mann–Whitney *U*‐test was applied for comparisons between two treatment conditions when the data were not normally distributed, as determined by the D'Agostino–Pearson normality test. Results were considered statistically significant at *P* < 0.05. Symbols *, **, *** and **** indicate *P*‐values of < 0.05, < 0.01, < 0.001, and < 0.0001, respectively. Details on the specific statistical analyses used for sequencing data are discussed in the relevant sections. The specific tests performed are listed in the figure legends.

## 3 Results

### 3.1 Characterization of primary human glioblastoma cell populations and verification of connexin 43 expression

**Fig. 1:**
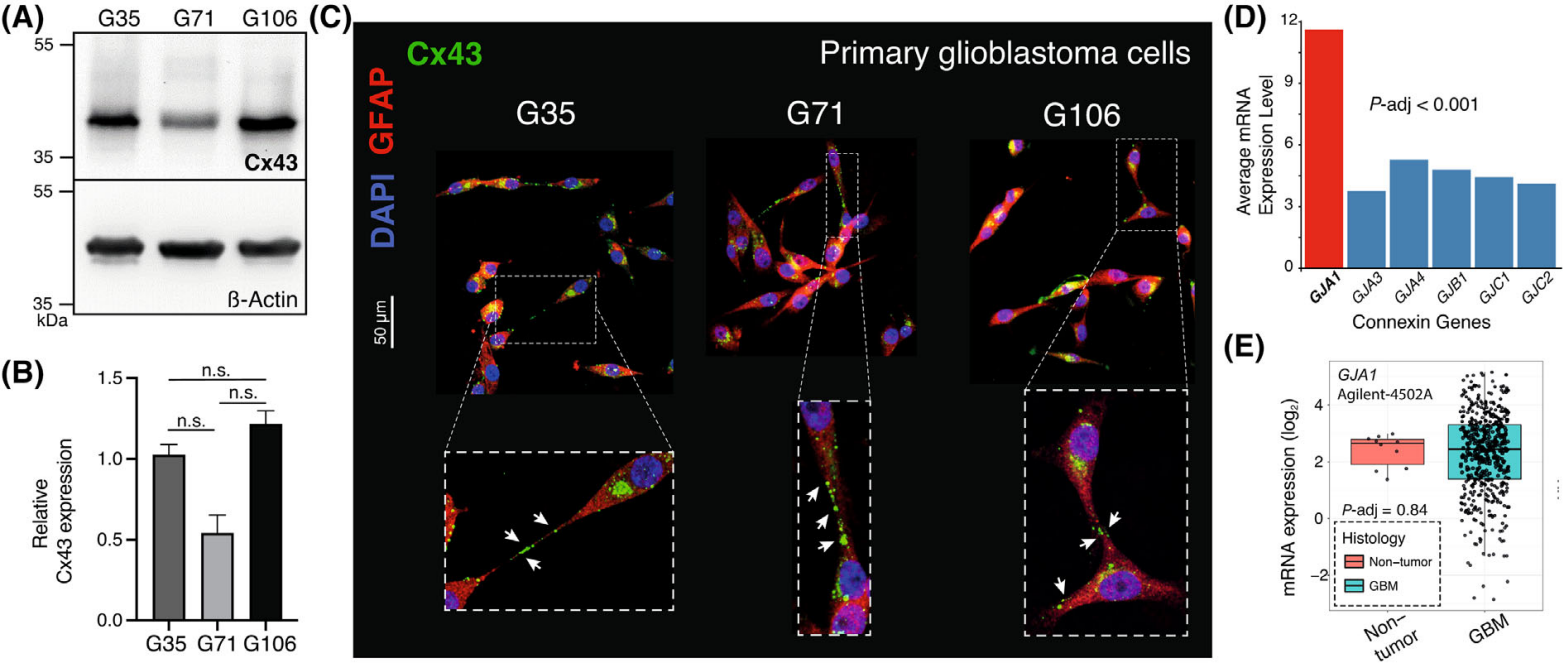
Positive *GJA1*/Cx43 expression in primary glioblastoma. (A) Verification of Cx43 protein expression using western blot analysis (*n* = 3). western blot was cropped to enhance clarity. (B) Densitometric quantification of Cx43 protein expression in relation to ß‐Actin expression levels from western blot analysis (*n* = 3). Statistical analysis was performed using a one‐way ANOVA, mean ± SD is shown. (C) Representative immunofluorescence images of differentiated primary glioblastoma cells (G35, G71 and G106, each *n* = 3). White arrows point to characteristic Cx43 expression along TMs. Scale bar: 50 μm. (D) Analysis of the mRNA expression level of genes encoding for different connexins using Affymetrix expression array HG‐U133A (*n* = 538). *GJA1* is encoding for the Cx43 protein (red bar), the other genes encode for Cx46, Cx37, Cx32, Cx45, Cx47 (blue bar). The *P*‐adj value for *GJA1* expression compared to other connexin genes was calculated using Dunnetts test. (E) Comparison of *GJA1* mRNA expression levels in *IDH*‐WT, *MGMT*‐methylated glioblastoma (*n* = 489) and non‐tumor tissue samples (*n* = 10) from the TCGA database Agilent‐4502A (*P* = 0.84). The *P*‐adj value was calculated using Tukey's Honest Significant Difference test. Cx43, connexin 43; GBM, glioblastoma; *GJA1*, gene encoding for Cx43; *P*‐adj, adjusted *P*‐value; TCGA, The Cancer Gene Atlas; TMs, tumor microtubes; WT, wild‐type.

Three primary human glioblastoma *MGMT* promoter hypermethylated cell populations (G35, G71, and G106) deduced from intraoperative surgical specimens were used for the following experiments. Expression of Cx43 was confirmed by immunoblotting of cell lysates of all three primary glioblastoma cell populations (Fig. 1A,B). Immunofluorescence imaging revealed Cx43 protein expression alongside interconnecting TM membranes indicating intercellular Cx43‐based GJ expression (Fig. 1C). Both in western blot analysis and immunofluorescence imaging, the G71 tends to exhibit lower Cx43 expression, even though statistical significance was not reached (Fig. 1B, Fig. S1A). For both methods, consistent cell confluences were used for all three cell populations to ensure reliability of the results (Fig. S1B).

Current literature and database research indicate that Cx43 is expressed in most glioblastomas and is the most highly expressed connexin among the group of connexins expressed in glioblastoma [40, 41] (Fig. 1D). However, analyses of multiple publicly available databases, including The Cancer Genome Atlas (TCGA, Agilent‐4502A and HG‐U133A dataset) [24], Rembrandt [25], and Gravendeel [26] reveal that at the messenger RNA (mRNA) expression level, Cx43 levels are not significantly different from those in non‐tumor tissue (Fig. 1E, Fig. S2). With our three primary glioblastoma cell populations, we identified suitable cells for further analysis, as Cx43 expression was observed at relevant levels in both western blot and immunofluorescence imaging.

### 3.2 Tonabersat functionally and morphologically disrupts tumor connectivity

**Fig. 2:**
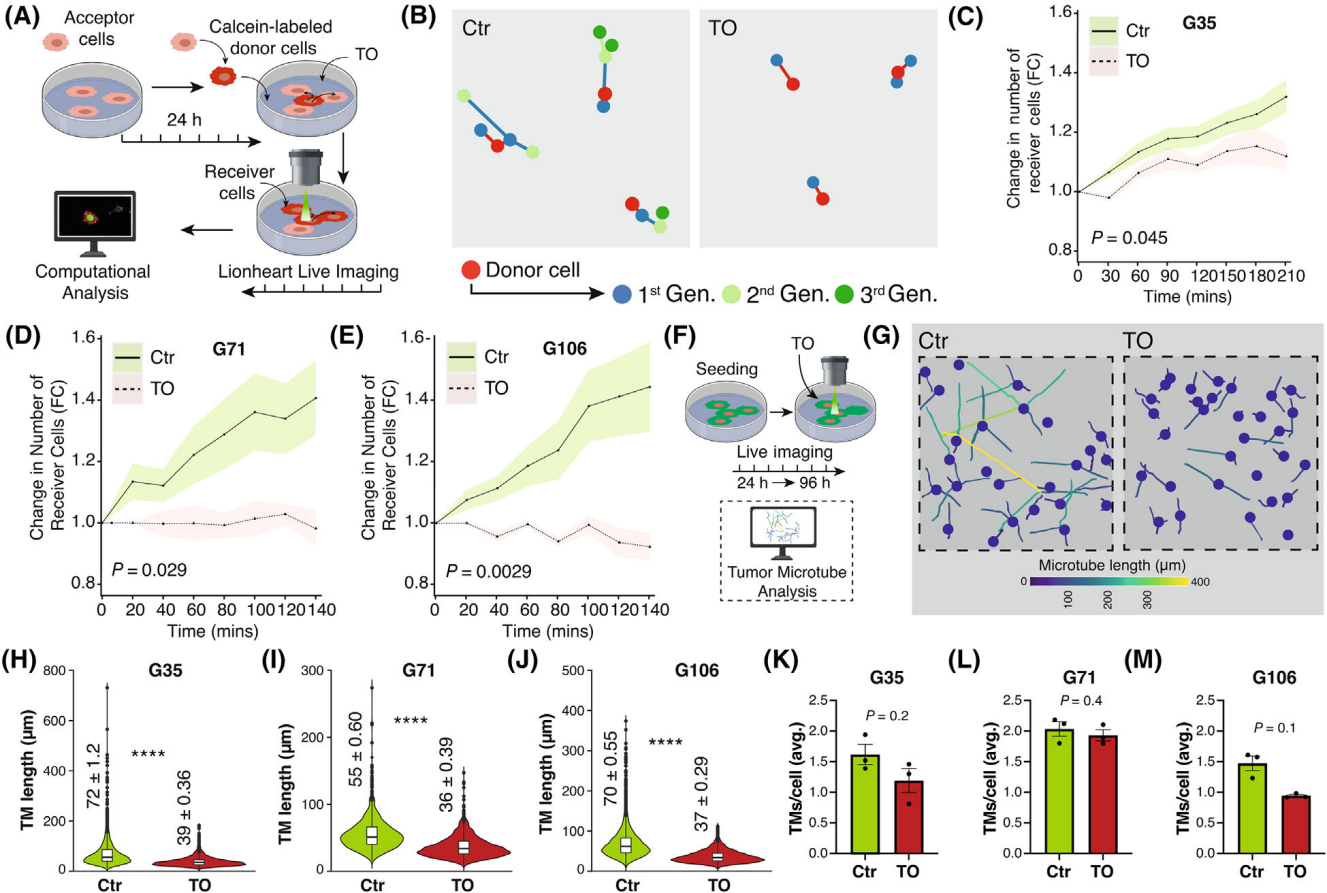
Tonabersat treatment results in a functional and morphological disconnection of glioblastoma cells. (A) Graphical illustration of the calcein dye transfer experiment. The number of receiver cells serves as a surrogate for the amount of intercellular traffic via gap junctions. (B) Illustration of calcein dye spreading from a single donor cell to multiple generations of receiver cells (*n* = 3 ROI per condition). (C–E) Quantification of the change in the number of receiver cells for G35 (C) (*n*(Ctr) = 3 vs. *n*(TO) = 3), G71 (D) (*n*(Ctr) = 6 vs. *n*(TO) = 3) and G106 (E) (*n*(Ctr) = 3 vs. *n*(TO) = 3). Statistical testing for significance was performed by a linear regression analysis. The points connected by a line represent the mean, while the shaded area indicates the SEM. (F) Workflow illustration for assessment of TM length and number. Measurement was performed 96 h after treatment with TO was started. (G) Color‐coded representation of TM length of G35 cells after treatment with TO. (H–J) Violin plot displaying the mean ± IQR as well as the TM length distribution for G35 (H), G71 (I) and G106 (J). The mean ± SEM are displayed numerically in the plot. (K–M) Barplot showing the average number of TMs per cell ± SEM for G35 (K), G71 (L) and G106 (M) 96 h after treatment. Statistical significance from analysis of three independent images was determined using the Mann–Whitney test for H–M. **** denote *P* < 0.0001. Avg, averaged; FC, Fold Change; Gen., generation; h, hours; IQR, interquartile range; mins, minutes; *P*‐adj, adjusted *P*‐value; TMs, tumor microtubes; TO, Tonabersat.

Cx43‐based GJs are known to interconnect glioblastoma cells to a functional multicellular network [2]. TO has previously been reported to inhibit GJ‐mediated communication between neurons and satellite glial cells in the trigeminal ganglion [42]. Here, we tested whether TO is able to prevent intercellular cytosolic traffic in glioblastoma cells measured by the transfer of calcein, a fluorescent molecule that exclusively spreads from cell‐to‐cell via intercellular GJs (Fig. 2A). Live cell imaging over a 4 h' time course showed that a single donor cell can not only transfer the dye to one receiver cell but that receiver cells can also function as “donor cells” and further spread the dye to multiple subsequent generations of receiver cells (Fig. 2B). Treatment with TO significantly reduced the increase in the number of receiver cells in all three cell populations by inhibiting the transfer of calcein across the GJs (FC for G35 after 210 min: 1.32 ± 0.05 (Ctr) versus 1.12 ± 0.04 (TO), *P* = 0.045; G71 after 140 min: 1.41 ± 0.12 (Ctr) versus 0.98 ± 0.06 (TO), *P* = 0.029; G106 after 140 min: 1.44 ± 0.15 (Ctr) versus 0.92 ± 0.04 (TO), *P* = 0.0029) (Fig. 2C–E).

Pharmacological blocking of GJs has previously been related to changes in tumor cell morphology [29]. To assess potential morphological alterations induced by TO treatment in tumor networks, live cell imaging was conducted 24 h after cell seeding, followed by additional 96 h of treatment with TO (Fig. 2F,G). G35 and G106 cell populations exhibited the longest TMs on average (mean ± SEM, G35: 72 ± 1.2 μm, G106: 70 ± 0.55 μm), followed by G71 cell populations (G71: mean ± SEM: 55 ± 0.6 μm) (Fig. 2H–J). G35 cells also had the highest number of ultra‐long TMs. Treatment with TO resulted in a significant reduction in the mean TM length across all three cell populations (Fig. 2G–J). The relative reduction in TM length was most pronounced in G35 cells, where the mean TM length decreased from 72 ± 1.2 μm to 39 ± 0.36 μm (mean ± SEM, *P* < 0.0001). Nonetheless, a significant reduction was also observed in G71 cells (55 ± 0.6 μm (Ctr) vs. 36 ± 0.39 μm (TO), *P* < 0.0001 and G106 cells [70 ± 0.55 μm (Ctr) vs. 37 ± 0.29 μm (TO), *P* ≤ 0.0001]. Significant differences in the mean TM length between untreated and TO‐treated cells were already seen after 48 h of treatment (Fig. S3A–C). Interestingly, the average number of TMs per cell remained largely unchanged in all three populations 96 h after treatment (G35: 1.6 ± 0.2 (Ctr) vs. 1.2 ± 0.2 (TO), *P* = 0.2; G71: 2.0 ± 0.1 (Ctr) vs. 1.9 ± 0.1 (TO), *P* = 0.4; G106: 1.5 ± 0.1 (Ctr) vs. 0.9 ± 0.02 (TO), *P* = 0.1; mean ± SEM) (Fig. 2K–M). Similarly, no difference in the number of TMs under TO treatment was observed after 48 h of treatment (Fig. S3D–F).

### 3.3 Tonabersat sensitizes glioblastoma cells to temozolomide‐induced cytotoxic effects

**Fig. 3:**
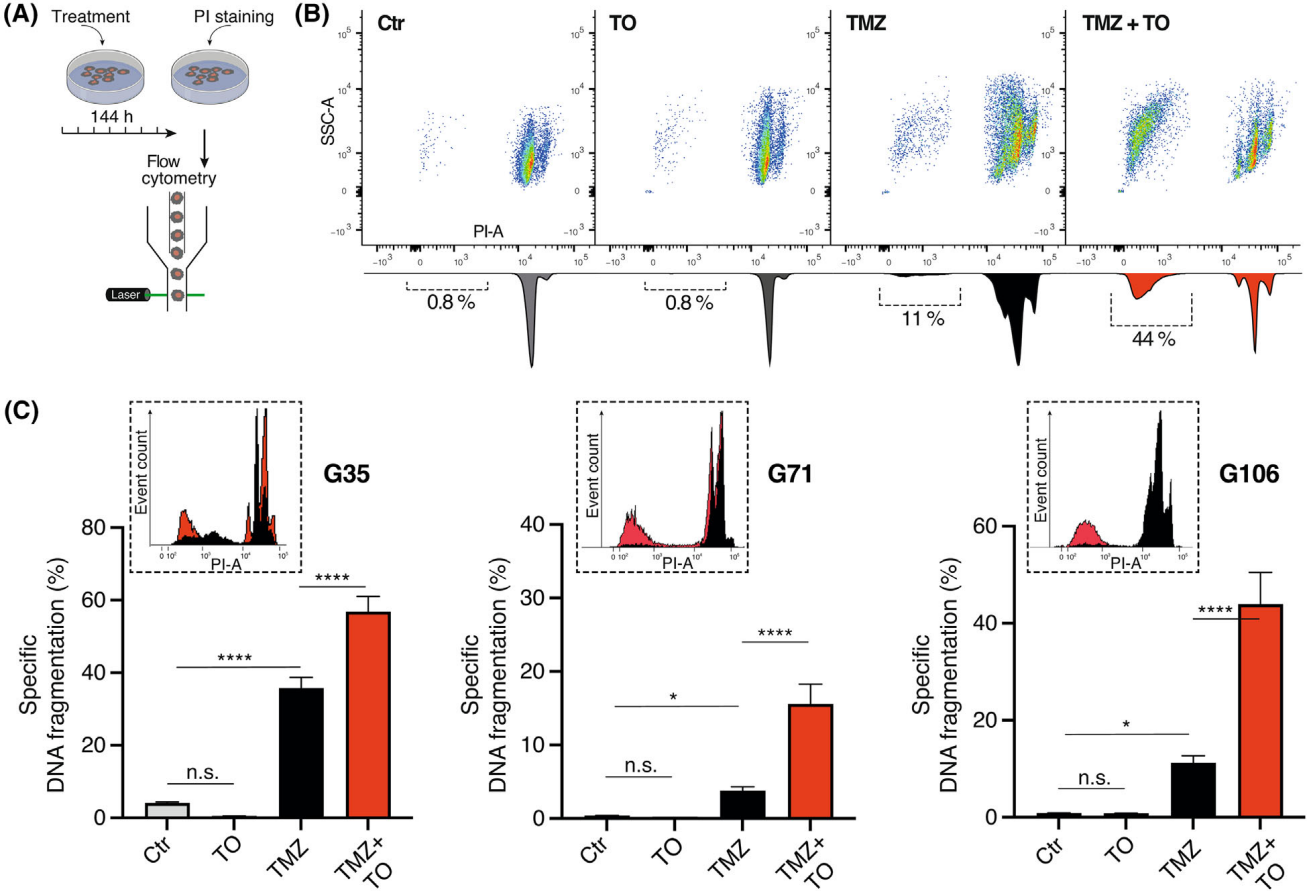
Tonabersat sensitizes glioblastoma cells to temozolomide‐induced cell death. (A) Workflow illustration of flow cytometric measurement of cell death. Using flow cytometry DNA fragmentation of PI‐stained nuclei was measured as a surrogate for cell death (indicated by the SubG1 peak). (B) Representative density plots and histograms from flow cytometric analysis of G106 cell population. The SubG1 peak is accentuated within the histograms and mean specific DNA fragmentation rate is depicted below. (C) Barplots showing the percentage of DNA fragmentation (mean ± SEM) for G35 (*n* = 6), G71 (*n* = 13) and G106 (*n* = 9) cell population. Representative histograms for single treatment with TMZ (black) and combination treatment of TMZ + TO (red) are depicted within the box at the top left of each barplot. Statistical significance was assessed using one‐way ANOVA testing. * and **** denote *P* < 0.05 and *P* < 0.0001. h, hours; PI, propidium iodide; PI‐A, propidium iodide‐area; TMZ, temozolomide; TO, Tonabersat.

Considering that intercellular communication within the syncytial tumor network is linked to tumor growth and therapy resistance [2, 5], the observed functional and morphological decoupling of glioblastoma cells through TO treatment may render the cells more susceptible to chemotherapeutic treatment. To test this, cells were treated for 144 h with TO, TMZ or a combination of both drugs, and DNA fragmentation of propidium iodide (PI)‐stained nuclei was measured by flow cytometry as a surrogate for cell death (Fig. 3A,B). TMZ concentration was chosen after performing a MTT analysis (Fig. S4). The analysis using both non‐linear and linear regression models revealed half maximal inhibitory concentration (IC50)‐values slightly above 50 μm for G35 and G106. For G71, higher values of almost 100 μm were observed, which is higher than physiologically achievable [43]. Given the physiological relevance and previous results of our group, we selected a concentration of 50 μm for TMZ treatment, which corresponds to levels detected in the interstitium of TMZ‐treated patients [3, 29, 43].

Treatment with TO alone did not lead to an increase in cell death, as measured by specific DNA fragmentation using flow cytometry. As expected, treatment with TMZ showed a significant increase in cell death rates compared to untreated control (specific DNA fragmentation for G35: 4.1% ± 0.25 (Ctr) vs. 36.0% ± 2.9 (TMZ), *P* < 0.0001; G71: 0.33% ± 0.07 (Ctr) vs. 3.8% ± 0.55 (TMZ), *P* = 0.018; G106: 0.48% ± 0.05 (Ctr) vs. 11.0% ± 1.5 (TMZ), *P* = 0.015). However, TO sensitized glioblastoma cells to TMZ‐induced cell death. In case of G71 and G106 cell populations, the sensitizing effect was about 4‐times higher than the amount of TMZ‐induced cell death rates (mean ± SEM; G71: 3.8% ± 0.55 (TMZ) vs. 16.0% ± 2.7 (TMZ + TO), *P* < 0.0001; G106: 11.0% ± 1.5 (TMZ) vs. 44.0% ± 6.6 (TMZ + TO), *P* < 0.0001) (Fig. 3C). Based on the DNA fragmentation rates, Bliss synergy was found for the combination treatment of TO and TMZ. The observed drug effects in case of combination treatment were up to 3.9‐times higher than the expected effects under the Bliss independent zero‐interaction hypothesis (G35: 1.6‐; G71: 3.9‐ and G106: 3.2‐fold higher).

### 3.4 Tonabersat‐mediated effects on cell proliferation

**Fig. 4:**
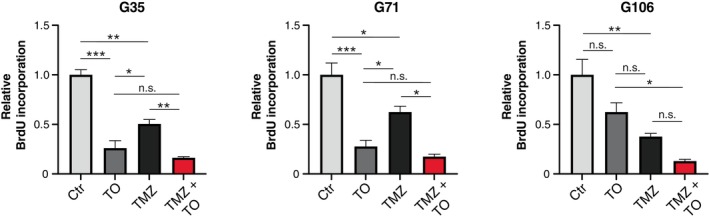
Tonabersat‐mediated effects on cell proliferation. The amount of incorporated BrdU was measured as a readout for cell proliferation. Depicted barplots show the relative BrdU incorporation rates measured 144 h after the start of treatment with TMZ, TO and the combination of TMZ and TO. Mean ± SD is depicted (*n* = 3). Statistical significance was determined using the one‐way ANOVA test. *, ** and *** denote *P* < 0.05, *P* < 0.01 and *P* < 0.001. BrdU, Bromodeoxyuridine; h, hours; TMZ, temozolomide; TO, Tonabersat.

To unveil the effect of TO on cell proliferation, the incorporation of bromodeoxyuridine (BrdU) into replicating DNA during the S phase of the cell cycle was measured by spectrophotometry. Unlike the effect on cell death, single treatment with TO resulted in a significant inhibition of cell proliferation for G35 and G71 cell populations (Fig. 4). As expected, treatment with TMZ resulted in reduced proliferative capacity by approximately 50% in all three cell populations. Combinatory treatment with TMZ and TO resulted in a further reduced percentage of actively dividing cells in G35 and G71 cell population. The evaluation of Bliss independence demonstrated that the effects of combinatory treatment were predominantly additive (G35: 0.22; G71: 0.19; G106: 0.13) unlike the effects on cell death. In MTT analysis, we could also observe a dose‐ and time‐dependent reduction of relative cell viability for TMZ and TO single treatment which might at least for TO be explained by the reduced cell proliferation as TO single treatment did not show any effects on cell death (Fig. S4).

### 3.5 Connexin 43 knockout leads to functional and morphological network disconnection

**Fig. 5:**
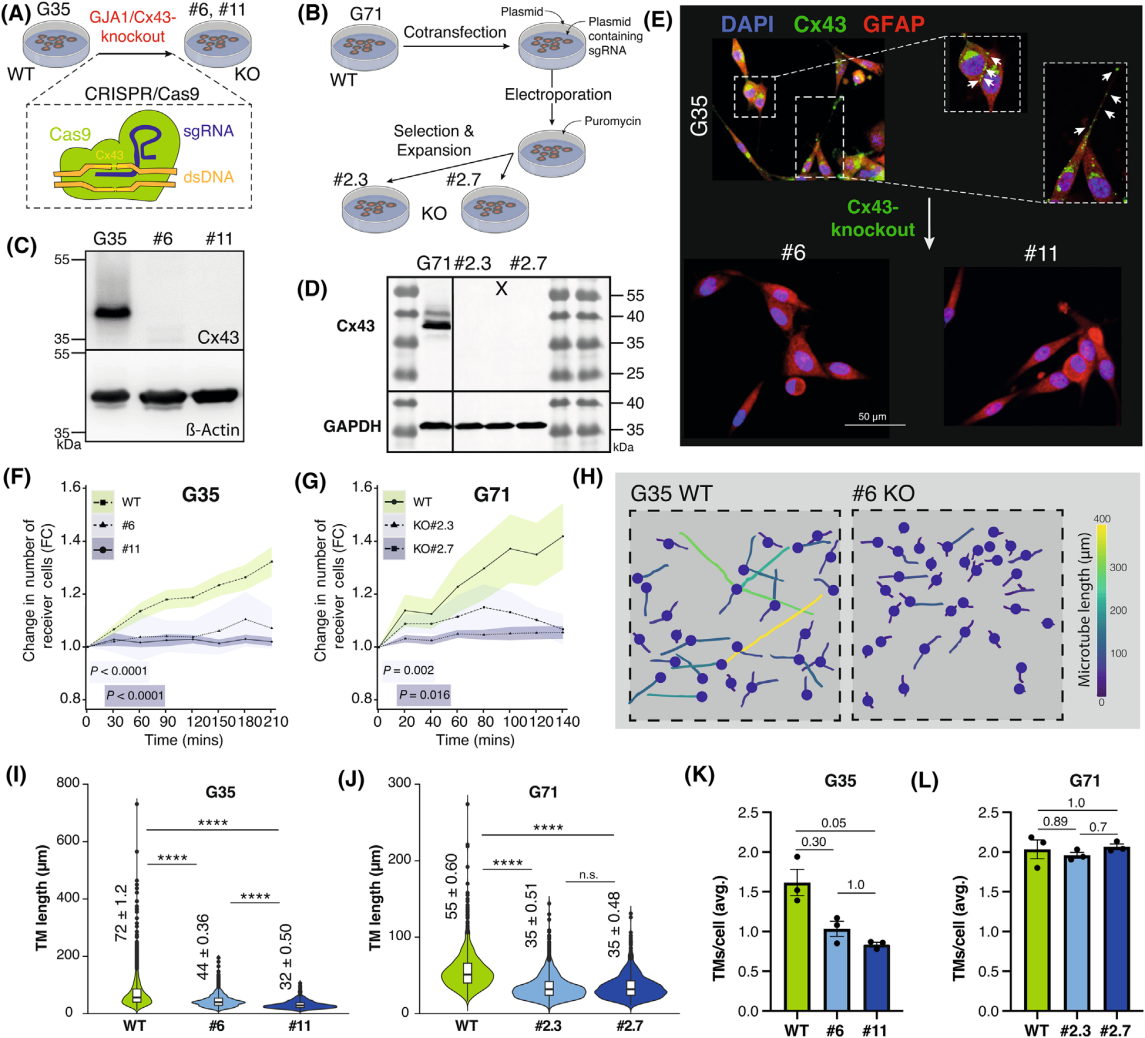
Connexin 43 knockout leads to functional and morphological network disconnection. (A, B) Workflow illustration of the CRISPR/Cas9‐based techniques used to generate the *GJA1*/Cx43 KO lines in G35 (A) and G71 (B). (C, D) Western blot analysis for Cx43 protein expression verifies the significant reduction of Cx43 protein levels for #6 and #11 (*n* = 2) (C) and #2.3 and #2.7 (*n* = 2) (D) knockout clones. Western blots were cropped to enhance clarity. A clone that was not utilized in the study is indicated with an X in (D). (E) Representative immunofluorescence images with Cx43 staining for #6 and #11 knockout clones and the corresponding Cx43 wild‐type cell population G35 (*n* = 3). Scale bar: 50 μm. (F, G) Quantification of the change of receiver cells for G35 (*n* = 3) and G71 (*n* = 6) as well as #6/#11 (*n* = 3) and #2.3/#2.7 (*n* = 7, *n* = 5) Cx43‐knockout clones in calcein dye transfer experiment. Statistical testing for significance was performed by a linear regression analysis. The points connected by a line represent the mean, while the shaded area indicates the SEM. (H) Color‐coded graphic illustration of TM length, shown for #6 KO clone. (I, J) Violin plot depicting the mean ± IQR as well as the distribution of TM length after treatment for 144 h. (K, L) Barplot depicting the average number of TM per cell. The mean ± SEM is displayed within the plot. Statistical significance from analysis of three independent images was assessed using one‐way ANOVA test with multiple comparisons for I–L. *P*‐adj are shown for K and L. **** denotes *P* < 0.0001. Avg., average; Cx43, connexin 43; dsDNA, double‐strand DNA; FC, Fold Change; *GJA1*, gene encoding for Cx43; hrs, hours; IQR, interquartile range; KO, knockout; mins, minutes; *P*‐adj, adjusted *P*‐value; sgRNA, Single guide RNA; TMs, tumor microtubes; WT, Wild‐type.

Our findings above show that TO not only diminishes cellular connectivity at both the morphological and functional level, but also sensitizes glioblastoma cells to TMZ‐induced toxicity. To investigate whether these effects are attributed to Cx43‐inhibition, we generated Cx43 KO cell clones of G35 and G71 cell populations to specifically eliminate Cx43 expression (Fig. 5A,B). Single cell colonies of G35 and G71 wild‐type (WT) cell populations were selected and analyzed for Cx43 expression by immunoblotting. Two stable Cx43 KO clones of G35 (#6 and #11) and of G71 cells (#2.3 and #2.7) showed an almost complete loss of Cx43 expression (Fig. 5C,D). Immunofluorescence staining of G35 WT cells and their KO clones also confirmed the lack of Cx43 expression in Cx43 KO cells while G35 WT cells showed the characteristic Cx43‐positive staining alongside TMs of interconnected cells (Fig. 5E). Live cell imaging for 4 h showed that almost no calcein dye was transferred from cell‐to‐cell in Cx43 KO clones of G35 and G71 cells, most likely due to the lack of GJs formed by Cx43 (Fig. 5F,G). Furthermore, the loss of Cx43 expression resulted in a significant decrease of characteristic ultra‐long TMs of WT G35 and G71 cells (Fig. 5H–J, Fig. S5A,B) and a reduction in mean TM length (Fig. 5I,J, Fig. S5C,D) in Cx43 KO cells. Again, the average number of TMs per cell remained mostly unchanged (Fig. 5K,L). Only in case of the G35 #11 KO clone, the average number of TMs per cell was reduced compared to the corresponding G35 WT cells (mean ± SEM, 0.8 ± 0.23 (#11 KO) vs. 1.0 ± 0.09 (G35 WT), *P* = 0.03) (Fig. 5K).

### 3.6 Network disconnection in connexin 43 knockout cells culminates in enhanced temozolomide‐mediated cell death rates

**Fig. 6:**
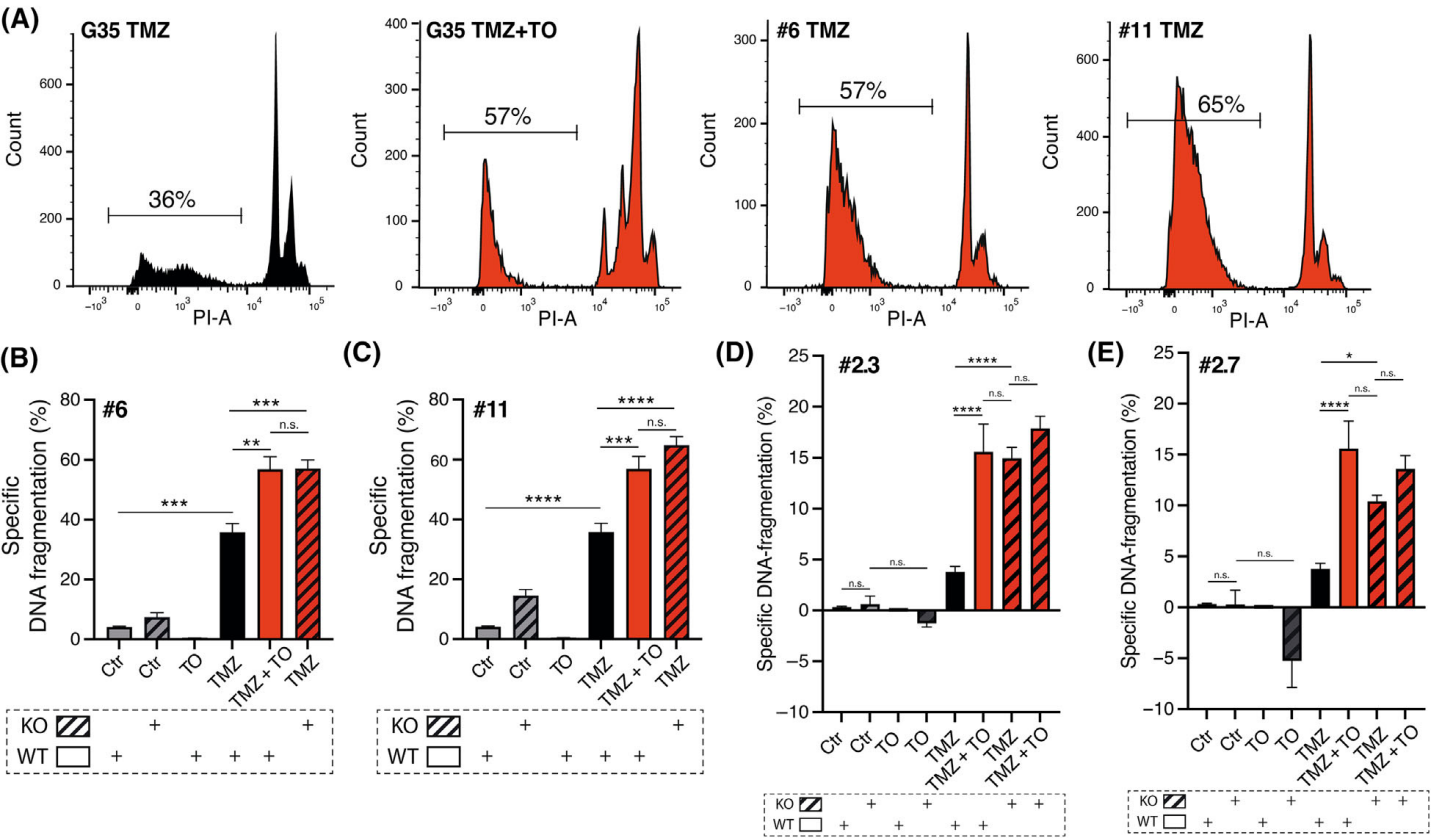
Network disconnection via connexin 43 knockout culminates in enhanced temozolomide‐mediated cell death rates. (A) Representative histograms from flow cytometric analysis of G35 WT cells and #6 and #11 KO clones for indicated conditions after treatment for 144 h. The SubG1 peak is highlighted in the histograms, and the average percentage of specific DNA fragmentation rate is shown. (B–E) Specific DNA fragmentation rates as a surrogate for cell death shown for G35 WT cells and #6/#11 KO clones (*n* = 6/*n* = 9) (B, C) as well as G71 WT cells and #2.3 and #2.7 KO clones (*n* = 13 and *n* = 4) (D, E). Mean ± SEM is depicted. Statistical significance was determined using the two‐way ANOVA test. *, **, *** and **** denote *P* < 0.05, *P* < 0.01, *P* < 0.001 and *P* < 0.0001. Ctr, control; h, hours; KO, knockout; *P*‐adj, adjusted *P*‐value; PI, propidium iodide; PI‐A, propidium iodide‐area; TMZ, temozolomide; TO, Tonabersat; WT, wild‐type.

In a next step, DNA fragmentation of propidium iodide (PI)‐stained nuclei was assessed by flow cytometry in TMZ‐treated Cx43 KO cells and compared to corresponding WT cells treated with TO and TMZ for 144 h (Fig. 6A–E). Cx43 KO clones #6 and #11 from G35 WT cells showed similar DNA fragmentation rates when treated with TMZ compared to G35 WT cells treated with TO and TMZ (mean ± SEM, 57% ± 2.9 (#6, TMZ) and 65% ± 2.8 (#11, TMZ) vs. 57% ± 4.2 (G35 WT, TMZ + TO), *P* = 1.0 and *P* = 0.32) (Fig. 6B,C). Similar results were observed for G71 KO cell clones #2.3 and #2.7 (mean ± SEM, 15% ± 1.1 (#2.3, TMZ) and 10% ± 0.61 (#2.7, TMZ) vs. 16% ± 2.7 (G71 WT, TMZ + TO), *P* = 1.0 and *P* = 0.35) (Fig. 6D,E). Significant differences were found between TMZ treatment in KO clones and wild‐type cells across all KO clones [mean ± SEM, 57% ± 2.9 (#6, TMZ) and 65% ± 2.8 (#11, TMZ) vs. 36% ± 2.9 (G35 WT, TMZ), *P* < 0.0001 and *P* < 0.0001; mean ± SEM, 15% ± 1.1 (#2.3, TMZ) and 10% ± 0.61 (#2.7, TMZ) vs. 3.8% ± 0.55 (G71 WT, TMZ), *P* < 0.0001 and *P* = 0.012] (Fig. 6B–E). Interestingly, additional TO treatment did not lead to increased cell death rates compared to TMZ treatment alone in KO clones #2.3 and #2.7. Therefore, the synergistic effect of TO with TMZ, induced pharmacologically, closely resembled that observed in Cx43 KO cells. These findings indicate that Cx43‐based GJs and their mediated intercellular communication contribute substantially to the reduced susceptibility of glioblastoma cells to TMZ. Furthermore, these findings imply that the effects of TO treatment were indeed attributed to the inhibition of Cx43‐based intercellular GJs, consequently augmenting the vulnerability of glioblastoma cells to chemotherapy.

### 3.7 Tonabersat enhances the temozolomide‐induced cascade of events leading to growth arrest and apoptosis

**Fig. 7:**
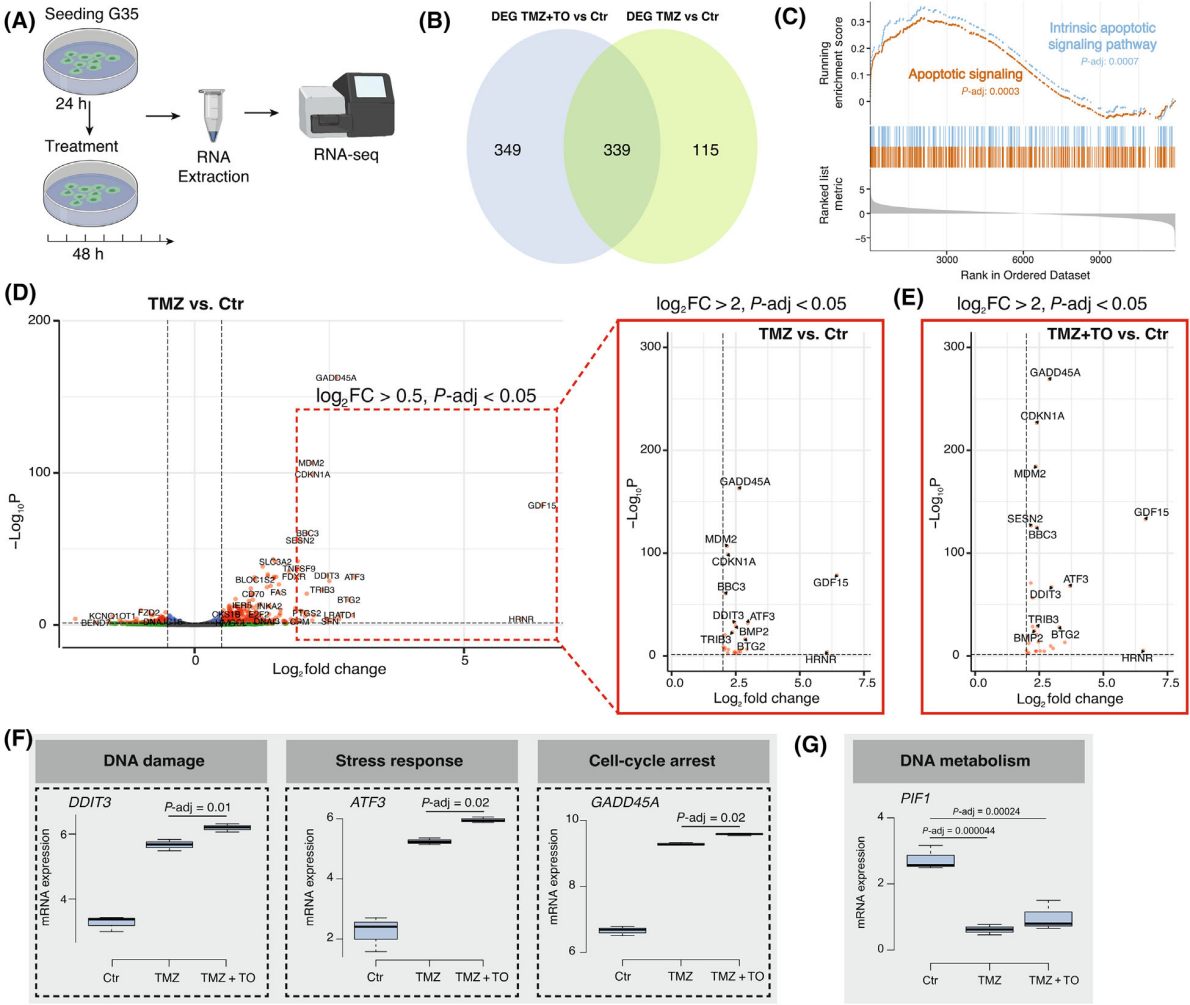
Tonabersat enhances the upregulation of temozolomide‐induced genes involved in the regulation of DNA damage, stress response and apoptosis. (A) Graphical abstract for 3′‐mRNA sequencing performed with G35 cells (*n* = 3, technical triplicate). (B) Venn diagram depicting number of differentially expressed genes under indicated treatment conditions (*P*‐adj < 0.05, log2FC > 0.5). (C) GSEA for upregulated pathways under TMZ + TO vs. TMZ treatment. (D) Volcano plot showing the DEGs for TMZ treatment (left graph in box with dashed red line: TMZ vs. Ctr, Log2FC > 0.5, *P*‐adj < 0.05; right graph in box with red line: TMZ vs. Ctr, Log2FC > 2, *P*‐adj < 0.05). Wald test was used for hypothesis testing with Benjamini‐Hochberg for multiple correction testing. (E) Volcano plot showing the DEGs for TMZ + TO vs. Ctr (Log2FC > 2, *P*‐adj < 0.05). Combined treatment with TMZ + TO shows almost the identical profile. (F) Boxplots depicting the relative expression of *DDIT3*, *ATF3* and *GADD45A* as representative genes for the regulation of DNA damage, stress response and cell cycle arrest. (G) Boxplot depicting the relative expression of PIF1. A pairwise *t*‐test was conducted to assess significance levels in “F, G,” based on three samples per treatment. The box represents the median ± IQR. Ctr, control; DEGs, differentially expressed genes; GSEA, gene set enrichment analysis; h, hours; Log2FC, log2 Fold Change; *P*‐adj, *P*‐adjusted; TMZ, temozolomide; TO, Tonabersat.

To evaluate the molecular processes underlying the TO‐enhancing effect of TMZ‐mediated toxicity, we performed 3′‐mRNA sequencing of primary G35 glioblastoma cells (Fig. 7A). TMZ treatment resulted in 454 differentially expressed genes, while the combined treatment with TMZ and TO affected 688 genes. Of these, 339 genes were shared between both treatment conditions (Fig. 7B). Gene set enrichment analysis (GSEA) of the differentially expressed genes (DEGs) shared between TMZ and TMZ + TO‐treated cells revealed enrichment in pathways related to intrinsic apoptotic signaling pathway and apoptotic signaling (Fig. 7C). Treatment with TMZ resulted in the significant differential expression of 25 genes, defined by an adjusted *P*‐value less than 0.05 and an absolute log2 fold change of at least 2 (log2FC > 2) (Fig. 7D). Many of the upregulated genes are involved in apoptosis following DNA damage (*DDIT3, BBC3, MDM2, SESN2, BMP2, TRIB3*). Other groups of genes included cellular stress response genes (*ATF3, GDF15*) as well as cell cycle arrest genes (*GADD45, CDKN1A, BTG2*) (Fig. 7D). Combined treatment with TMZ + TO resulted in an almost identical profile of 29 significantly differentially expressed genes (log2FC > 2, *P*‐adj < 0.05), 22 of which were already found in TMZ‐treated cells, but with higher variance than the TMZ‐responsive genes (Fig. 7E). Apoptosis‐associated (*DDIT3, BBC3, MDM2, SESN2, BMP2, TRIB3*), stress response (*ATF3*, *GDF15*) and cell cycle arrest genes (*GADD45, CDKN1A* and *BTG2*) were among the most significantly upregulated genes and showed a much stronger gene activation effect than that of TMZ alone (Fig. 7F). Significantly less genes were found to be downregulated. The most downregulated gene in both TMZ and TMZ + TO‐treated glioblastoma cells was *PIF1* (Fig. 7G). Downregulation of *PIF1* has been shown to induce apoptosis and cell cycle arrest [44]. These data show that the combined treatment of glioblastoma cells with TMZ + TO induces a more powerful apoptotic response than TMZ alone, leading to strong activation of genes that ultimately execute DNA damage, cell cycle arrest and apoptotic cell death.

Furthermore, we conducted an analysis of the Agilent‐4502A dataset of the TCGA database to examine the correlation between *GJA1* expression (*GJA1*, gene encoding for Cx43) and the genes that showed to be significantly upregulated under therapy with TMZ and TO in our study (Fig. S6) [24]. The analysis revealed that some of these genes are indeed correlated with the *GJA1* expression. Especially, *GADD45A*, *CDKN1A*, and *BTG2* show a positive correlation with *GJA1* expression (*GADD45A*, *CDKN1A*, and *BTG2*: *P* < 0.0001; *GADD45A*: *r* = 0.21, *CDKN1A*: *r* = 0.15, *BTG2*: *r* = 0.09). Furthermore, *BMP2* shows a negative correlation with *GJA1*/Cx43 expression (*P* < 0.0001, *r* = −0.27) and *GDF15* shows a positive correlation with *GJA1* expression (*P* = 0.03, *r* = 0.1). These findings suggest that *GJA1* expression levels are intricately linked with the regulation of genes involved in key cellular processes.

## 4 Discussion

In this study, we demonstrate that pharmacological inhibition of GJ with TO resulted in a breakdown of intercellular connectivity in glioblastoma. This breakdown was reflected by an inhibition of intercellular cytosolic traffic via GJs on the functional level and a significant reduction in the length of TMs on the morphological level. TO‐mediated inhibition of intercellular connectivity was accompanied by a sensitizing effect on TMZ‐induced cell death. The TO‐induced synergistic effects with TMZ were similar to those seen in Cx43 KO glioblastoma cell clones treated with TMZ alone suggesting an important role of TM localized Cx43 in limiting TMZ‐mediated cell death. Moreover, GSEA revealed that TO‐mediated GJ inhibition together with TMZ treatment profoundly activated genes that are associated with DNA damage, stress response, cell cycle arrest and regulation of apoptosis.

The role of Cx43 in tumor pathogenesis is more complex than mere expression levels, and opposing roles have been described [10, 45, 46, 47]. Although literature shows that Cx43 can act as both a tumor suppressor [48, 49, 50] and a tumor promoter [51, 52] in cancer, most studies suggest that Cx43 is rather tumor‐promoting in glioblastoma [53, 54]. In the context of glioblastoma, Cx43 has been associated with tumor invasion and migration [45, 55, 56] as well as resistance to treatments such as temozolomide [5, 41, 57] and radiation [2]. It has also been linked to poor survival outcomes [41]. These effects are partly attributable to Cx43 as a key component for the formation of malignant connectivity by interconnecting tumor cells into a multicellular network [2, 29]. Therefore, targeting Cx43 and GJ through various mechanisms is considered a promising therapeutic approach in glioblastoma [3, 29, 41, 54, 58].

The association between Cx43 and network formation has also been demonstrated in several other brain tumor entities [1, 2, 5]. Intercellular communication through GJs for instance enables brain metastatic cancer cells to transfer the second messenger cGAMP to astrocytes [51]. This results in an activation of the STAT1 and NF‐kB pathway thereby supporting tumor growth and resistance to chemotherapy.

TO has been shown to reduce ATP release from Cx43 hemichannels by directly blocking the channel and inhibiting GJ communication from cell‐to‐cell through coupled GJs in human cerebral microvascular endothelial cells [13]. Moreover, an inhibition of GJ coupling between neurons and glial cells had been shown for TO previously [42].

Building on these findings from previous investigations which demonstrated the inhibition of GJ coupling by administration of TO, we found that TO significantly reduced intercellular cytoplasmic transfer of calcein via GJs and shortened the length of TMs in patient‐derived primary glioblastoma cells. Similar properties have already been observed with the GJ modulator MFA. Multiple studies have shown that MFA functionally blocks GJs and reduces GJ‐mediated intercellular exchange in glioblastoma cells [29, 59, 60]. Furthermore, in xenograft and immunocompetent models, both MFA and TO have also been shown to restrict progression of brain metastases [51]. However, it remains unclear for MFA whether the substance crosses the blood–brain barrier. A multicenter phase I/II trial of MFA/TMZ combination therapy in relapsed *MGMT*‐methylated glioblastoma (MecMeth/NOA‐24 trial, EudraCT2021‐000708‐39) which was initiated upon our previous experimental findings will measure the concentration of MFA within the resected tumor tissue to address this question and will for the first time evaluate the safety and feasibility of a therapeutic approach directed against the tumor network [29, 61]. However, TO has intensively been studied in several clinical trials as a potential treatment or prophylactic treatment for migraine and administered orally to over 1000 humans [18]. Unlike MFA, TO is clearly capable of crossing the blood–brain barrier, making it a more suitable candidate for a translation into clinical practice [12, 18]. Meulenaere et al. and Zoteva et al. [15, 16] demonstrated the potential of TO as an adjuvant GJ‐directed therapy for glioblastoma in a preclinical F98 rat model showing a significant reduction in tumor volumes in rats treated with TO in combination with radiation and TMZ treatment [15]. Zoteva et al. further showed increased median survival rates when TO was added to the standard regimen of radiation and TMZ [16]. These findings provide the first *in‐vivo* proof of concept for the therapeutic potential of TO in glioblastoma treatment, though no functional analyses on TO effects were performed.

Previous work has provided important insight into the tumorigenic role of Cx43 and its involvement in the formation of tumor networks. A recent study using a CRISPR/Cas9 system to knockout Cx43 in breast cancer cell lines showed that Cx43 modifies the formation of tunneling nanotubes (TNTs) and facilitates intercellular communication. Cx43 KO resulted in a significantly reduced number and length of TNTs [62]. Furthermore, in glioblastoma cells, a short hairpin RNA‐mediated knockdown of *GAP43*, which is particularly relevant for the formation of neurite‐like membrane protrusions in neural and non‐neural cells, has shown a reduction of Cx43 GJ protein expression and intercellular TM connections [2]. Consistently, we found that a CRISPR/Cas9‐mediated knockout of Cx43 in glioblastoma cells led to a profound reduction in the TM length and decrease of intercellular calcein dye transfer, supporting the important stabilizing role of Cx43 GJ‐mediated communication in the tumor network.

Given that tumor networks and Cx43‐mediated intercellular communication play pivotal roles in tumor growth and resistance to anticancer therapy, the susceptibility of tumor cells to chemotherapy is increased when their connectivity is disrupted [5]. Previous investigations used a CRISPR/Cas9‐mediated knockout of Cx43 to demonstrate the successful sensitization of lung cancer cells to cisplatin [63]. In the present study, the combined treatment of the GJ inhibitor TO together with TMZ profoundly sensitized glioblastoma cells to TMZ‐mediated cell death. Similarly, our Cx43 KO glioblastoma cell clones exhibited an increased vulnerability to TMZ single treatment, showing that the TO‐enhancing cytotoxic effects for TMZ is most likely facilitated by TO‐mediated inhibition of GJs formed by Cx43.

On the molecular level, all three cell cycle arrest genes that were upregulated under combination therapy in our study (*GADD45A*, *CDKN1A*, and *BTG2*), where Cx43 function was inhibited, showed a positive correlation with *GJA1* levels in brain tumor tissue. Similarly, *GDF15*, which is associated with the cellular stress response, also showed a positive correlation with *GJA1*. This suggests that glioblastomas with high *GJA1* expression might have an enhanced ability to pause the cell cycle or activate stress response mechanisms, potentially contributing to chemoresistance, where cancer cells become less sensitive to treatment. The negative correlation between *GJA1* and *BMP2* implies that high Cx43 levels are associated with lower levels of cell death. These findings align with observations that high Cx43 levels might be linked to poorer survival outcomes [41]. Therefore, therapies that reduced Cx43 levels may promote apoptosis (as indicated by *BMP2*) and weaken mechanisms that enable cells to manage stress and pause the cell cycle for damage repair, as suggested by the associated decrease in the expression of genes like *GDF15*, *GADD45A*, *CDKN1A*, and *BTG2*. It is important to note the limitation in directly comparing this with tumors that intrinsically have low *GJA1* levels. The biological context of an intrinsic low level of *GJA1* in untreated brain tumor tissue may differ significantly from a therapy‐induced reduction in *GJA1*. However, based on these observations, the TCGA data aligns with and supports our therapeutic approach of targeting Cx43‐based GJs.

Interestingly, GSEA analyses revealed that apoptotic signaling pathways were most highly upregulated by the combinatorial treatment with TMZ and TO compared to TMZ single treatment. Strikingly, a number of the significantly upregulated genes are well‐known, high‐confidence p53 target genes induced following DNA damage [64, 65]. As such, p53 transactivates proapoptotic genes, such as *DDIT3, BBC3, GDF15, SESN2, MDM2*, as well as genes that induce cell cycle arrest, such as *GADD45, CDKN1A and BTG2*. Other significantly upregulated genes in TMZ and TMZ + TO‐treated glioblastoma cells may play dual roles. For instance, the transcription factor *ATF3* has been demonstrated to function as both a transcriptional activator and a repressor, it can either promote or suppress apoptosis and proliferation, two critical processes for cancer progression [66, 67]. Similarly, *TRIB3*, upregulated in both TMZ and TMZ + TO‐treated glioblastoma cells, plays an important role in proliferation and osteogenic differentiation but also has been shown to be involved in endoplasmatic reticulum stress‐dependent apoptotic cell death [68, 69]. The increased expression of MDM2 in TMZ and TMZ + TO‐treated cells is particularly striking because MDM2 and p53 together form a negative feedback control loop [70]. On one hand, MDM2 is an important negative regulator of p53 as it binds and inhibits p53 activity. On the other hand, *MDM2* is a p53‐responsive gene, meaning that its transcription can be activated by p53 resulting in higher *MDM2* expression. Upon DNA damage, p53 protein levels rapidly increase due to inhibition of *MDM2*/p53 interaction via several mechanisms [71]. The outcome of p53 activation in cells ranges from reversible cell cycle arrest and induction of DNA repair to more drastic responses, such as cell death by apoptosis or senescence [64]. Our data, alongside with the observation that combined treatment with TO and TMZ resulted in the highest proportion of dead cells in three different glioblastoma cell populations, are consistent with the induction of p53 pathways, with apoptosis being the major response to TMZ in glioblastoma cells. Furthermore, this highlights that TO significantly augments this response to TMZ on the molecular level.

Despite extensive research, the precise mechanisms behind the sensitizing effects of GJ‐targeted therapies to cytotoxic medication remain largely unknown. TO may enhance the toxicity of TMZ by uncoupling of tumor cells on a functional and morphological levels enabling higher TMZ concentrations within individual, uncoupled tumor cells. This, in turn, may sensitize the cells to TMZ‐induced cell death. Additionally, intercellular survival signaling through GJ formations between glioblastoma cells and nonmalignant astrocytes may contribute to the anti‐tumoral effects of GJ‐targeted therapies, as demonstrated in mouse models of brain metastasizing breast and lung cancer [2, 51]. Furthermore, GJ inhibition could lead to reduced transfer of pro‐survival noncoding RNA [53] or disruption of intracellular homeostasis through an imbalance of GJ‐permeable molecules like Ca^2+^ [5]. At the molecular level, TO may be significantly activating pathways involved in DNA damage control, response to cellular stress and regulation of apoptosis and cell death. Despite the fact that the precise mechanism by which TO increases TMZ‐mediated cell death in glioblastoma cells remains unknown, TO might represent a feasible candidate for the translation of a therapeutic approach directed against tumor networks in glioblastoma.

## 5 Conclusions

This study demonstrates that TO exerts inhibitory effects on Cx43‐based intercellular GJs in glioblastoma resulting in both a functional and a morphological breakdown of the malignant cell‐to‐cell connectivity. This breakdown was reflected by the inhibition of intercellular cytosolic traffic via GJs at the functional level and a significant reduction in the length of TMs at the morphological level. Furthermore, TO‐mediated inhibition of the malignant connectivity was accompanied by a reinforcement of chemotherapy‐induced antitumor effects. Considering the well‐tolerable side‐effect profile observed in previous clinical trials for migraine therapy, TO might harbor the potential of bridging the concept of a GJ‐targeted therapeutic approach from bench to bedside.

## Conflict of interest

UH received advisory board and speaker's honoraria from Medac and Bayer, and advisory board honoraria from Servier and Oncomagentx. The other authors declare no conflict of interests.

## Author contributions

Conceptualization and design: BOE, MS, A‐LP; Methodology: ENCS, BOE, PF‐P, M‐AW, MS, A‐LP; Resources: TP, PS and HV; Data acquisition ENCS, BOE, BEFP, AM, M‐CH, MR, HS, JR, TP, PS, MS, A‐LP; Software and bioinformatical analysis: BEFP, A‐LP; Visualization: ENCS, BEFP, MS, A‐LP; Writing—original draft: ENCS, BOE, MS, A‐LP; Writing—review and editing: ENCS, BOE, BEFP, AM, M‐CH, MR, HS, JR, TP, PS, PF‐P, M‐AW, MH, UH, HV, AW, MS, A‐LP; Supervision: M‐AW, MH, UH, HV, MS; Funding acquisition: MS.

### Peer review

The peer review history for this article is available at https://www.webofscience.com/api/gateway/wos/peer‐review/10.1002/1878‐0261.13786.

## Supporting information


**Fig. S1.** Immunofluorescence imaging for Cx43 expression.
**Fig. S2.** *GJA1* mRNA expression in publicly available datasets.
**Fig. S3.** Tonabersat treatment results in a reduced TM length.
**Fig. S4.** Relative cell viability after treatment with Temozolomide and Tonabersat.
**Fig. S5.** Connexin 43 knockout leads to a reduced TM length.
**Fig. S6.** Correlation between *GJA1* expression levels from Agilent‐4502A dataset of the TCGA database and genes upregulated under combined TMZ and TO therapy.
**Table S1.** Guide RNAs used for gene editing of the *GJA1* gene encoding for Cx43 for G35 cell population.
**Table S2.** Single guide RNA used for gene editing of the *GJA1*/Cx43 gene for G71 cell population.

## Data Availability

The authors declare that the data within this manuscript are available from the corresponding authors upon reasonable request.
