# Supplementary material for: Tonabersat enhances temozolomide‐mediated cytotoxicity in glioblastoma by disrupting intercellular connectivity through connexin 43 inhibition
Source: Mol Oncol. 2024 Dec 16;19(3):878–98. doi: 10.1002/1878-0261.13786 (PMC11887680; doi:10.1002/1878-0261.13786)
Supplement: Supplementary file 1 — Fig. S1. Immunofluorescence imaging for Cx43 expression. Fig. S2. GJA1 mRNA expression in publicly available datasets. Fig. S3. Tonabersat treatment results in a reduced TM length. Fig. S4. Relative cell viability after treatment with Temozolomide and Tonabersat. Fig. S5. Connexin 43 knockout leads to a reduced TM length. Fig. S6. Correlation between GJA1 expression levels from Agilent‐4502A dataset of the TCGA database and genes upregulated under combined TMZ and TO therapy. Table S1. Guide RNAs used for gene editing of the GJA1 gene encoding for Cx43 for G35 cell population. Table S2. Single guide RNA used for gene editing of the GJA1/Cx43 gene for G71 cell population. [file MOL2-19-878-s001.docx]

**Supplementary Material**

**Tonabersat enhances temozolomide-mediated cytotoxicity in glioblastoma by disrupting intercellular connectivity through connexin 43 inhibition**

Elena N. C. Schmidt^1,2^*, Bernd O. Evert^3^*, Barbara E. F. Pregler^1,2^, Ahmad Melhem^1,2^, Meng-Chun Hsieh^1,2^, Markus Raspe^1,2^, Hannah Strobel^6^, Julian Roos^6^, Torsten Pietsch^4^, Patrick Schuss^1,5^, Pamela Fischer-Posovszky^6,7^, Mike-Andrew Westhoff^6^, Michael Hölzel^8^, Ulrich Herrlinger^9^, Hartmut Vatter^1^, Andreas Waha^4^, Matthias Schneider^1,2^, Anna-Laura Potthoff^1,2,4^

*^1^Department of Neurosurgery, University Hospital Bonn, 53127 Bonn, Germany*

*^2^Brain Tumor Translational Research Group, University Hospital Bonn, Germany*

*^3^Department of Neurology, University Hospital Bonn, 53127 Bonn, Germany*

*^4^Department of Neuropathology, University Hospital Bonn, 53127 Bonn, Germany*

*^5^Current address: Department of Neurosurgery, BG Klinikum Unfallkrankenhaus Berlin BG, 12683 Berlin, Germany*

*^6^Department of Pediatrics and Adolescent Medicine, University Medical Center Ulm, 89075 Ulm, Germany*

*^7^German Center for Child and Adolescent Health (DZKJ), partner site Ulm, 89075 Ulm, Germany*

*^8^Institute of Experimental Oncology, University Hospital Bonn, 53127 Bonn, Germany*

*^9^Department of Neurooncology, Center for Neurology and Center of Integrated Oncology ABCD, University Hospital Bonn, 53127 Bonn, Germany*

**shared first authorship*

**Supplementary Figure S1**


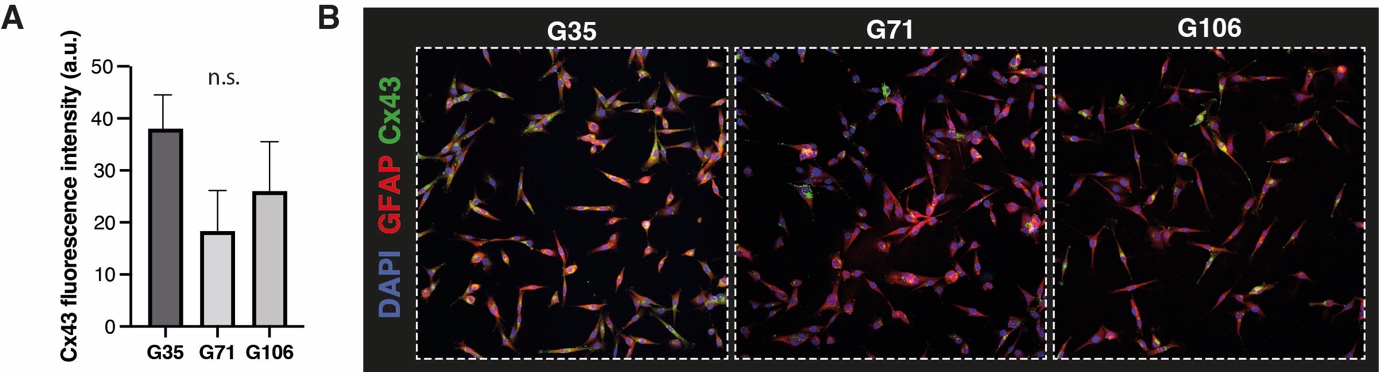


**Supplementary Figure S1: Immunofluorescence imaging for Cx43 expression**

(A) Quantification of Cx43 expression levels from immunofluorescence imaging of the three primary glioblastoma cell populations G35, G71 and G106 (n=3 each). (B) Three representative images are depicted to demonstrate consistent confluency across all three cell populations.

Abbreviations: a.u., arbitrary unit; Cx43, connexin 43.

**Supplementary Figure S2**


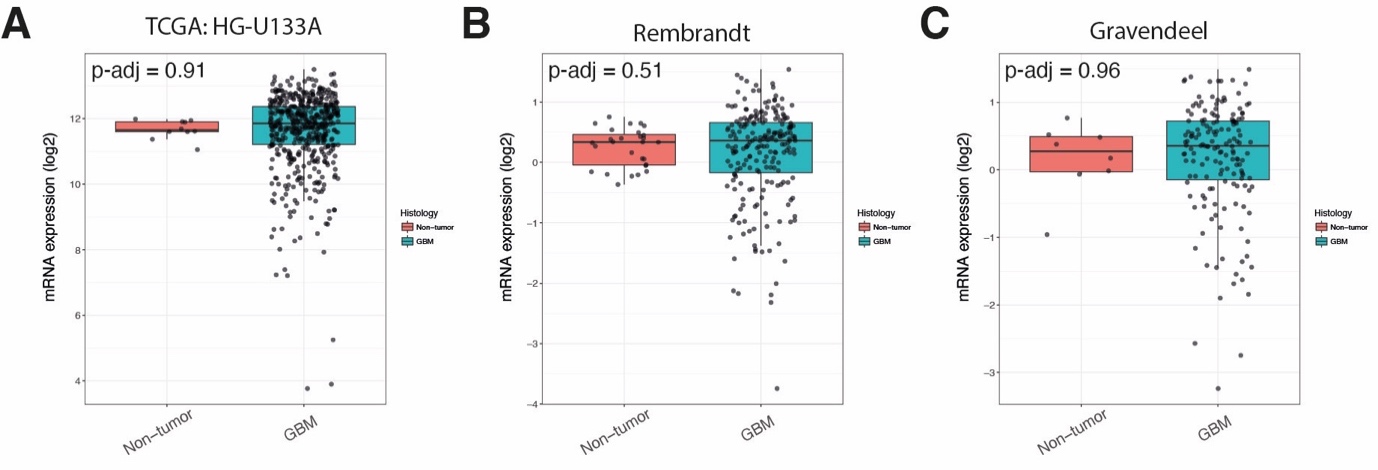


**Supplementary Figure S2: *GJA1* mRNA expression in publicly available datasets**

(A) Comparison of *GJA1* mRNA expression levels in *IDH*-wildtype, *MGMT*-methylated glioblastoma and non-tumor tissue samples from The Cancer Gene Atlas database HG-U133A. (B-C) Comparison of *GJA1* mRNA expression levels in the Rembrandt (B) and Gravendeel (C) dataset without further differentiation of glioblastoma samples by molecular subtypes. Adjusted p-values were calculated using Tukey's Honest Significant Difference test.

Abbreviations: GBM, glioblastoma; *GJA1*, gene encoding for Cx43; p-adj, adjusted p-value; TCGA, The Cancer Genome Atlas

**Supplementary Figure S3**

**
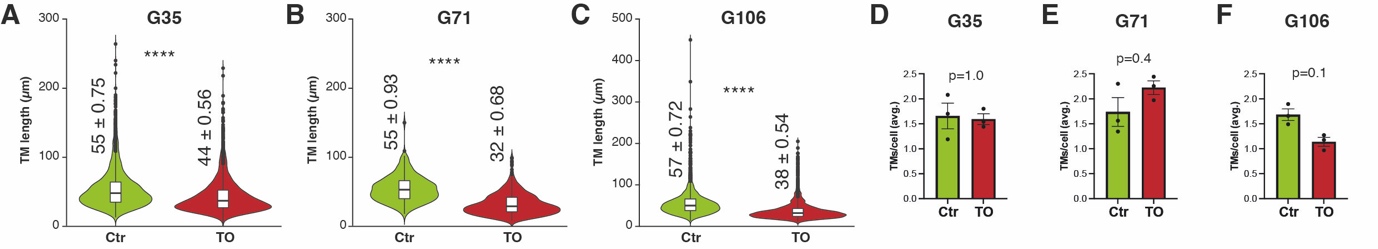
**

**Supplementary Figure S3: Tonabersat treatment results in a reduced TM length**

(A-C) Violin plot displaying the mean, IQR and TM length distribution 48 hours after treatment for G35 (A), G71 (B) and G106 (C). The mean ± SEM is displayed within the plot. (D-F) Barplot showing the average number of TMs per cell after 48 hours of treatment for G35 (D), G71 (E) and G106 (F). Statistical significance significance from analysis of three independent images was determined using the Mann-Whitney test. **** denote p<0.0001.

Abbreviations: Avg, averaged; Ctr, control; TM, tumor microtube; TO, Tonabersat.

**Supplementary Figure S4**


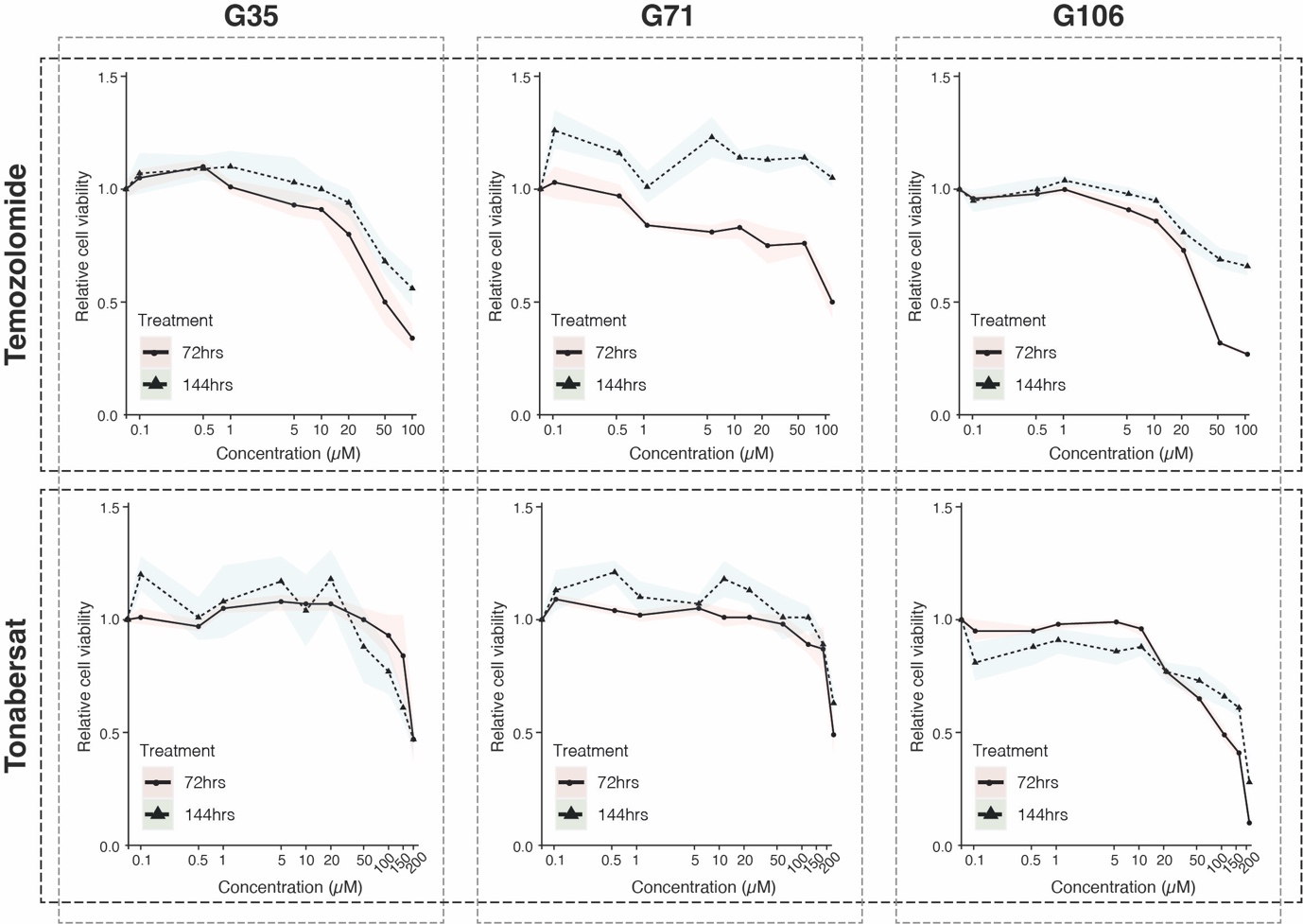


**Supplementary Figure S4: Relative cell viability after treatment with Temozolomide and Tonabersat**

Relative cell viability after treatment with Temozolomide (TMZ, upper row) and Tonabersat (TO, bottom row) is depicted for G35, G71 and G106. Each plot includes measurement after treatment for 72 hrs (red) and for 144 hrs (blue). For TMZ, concentrations ranging from 0.1 to 100 µM were used, while for TO, concentrations up to 200 µM were applied. Mean ± SEM is displayed.

Abbreviations: hrs, hours.

**Supplementary Figure S5**

**
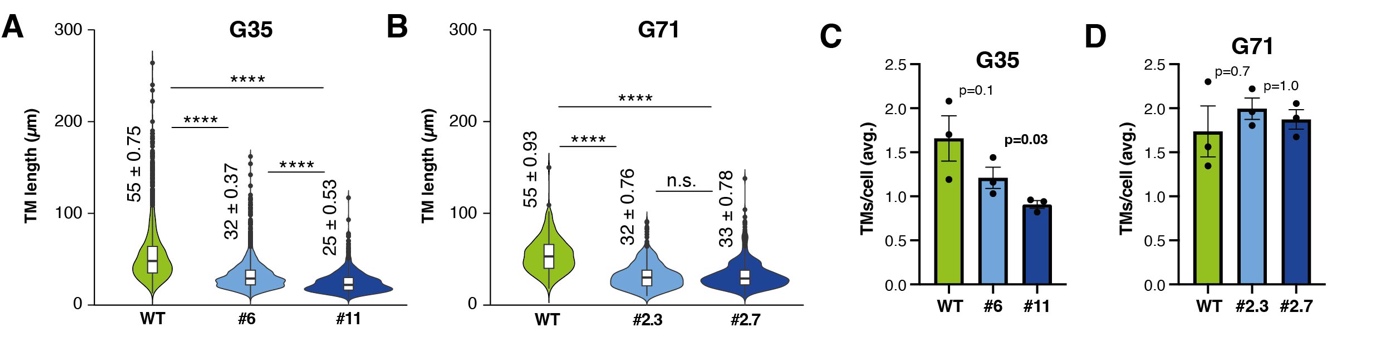
**

**Supplementary Figure S5: Connexin 43 knockout leads to a reduced TM length**

(A, B) Violin plot displaying the mean, IQR and TM length distribution after 48 hours for G35 (A) and G71 (B) WT cells and corresponding KO clones #6, #11 and #2.3, #2.7. The mean ± SEM is displayed within the plot. (C, D) Barplot showing the average number of TMs per cell for G35 (A) and G71 (B) and KO clones. Statistical significance significance from analysis of three independent images was determined using One-way ANOVA test with multiple comparisons. Adjusted p-values are shown for C and D. **** denotes p<0.0001.

Abbreviations: Avg, averaged; TM, tumor microtube; TO, Tonabersat; WT, Wildtype.

**Supplementary Figure S6**


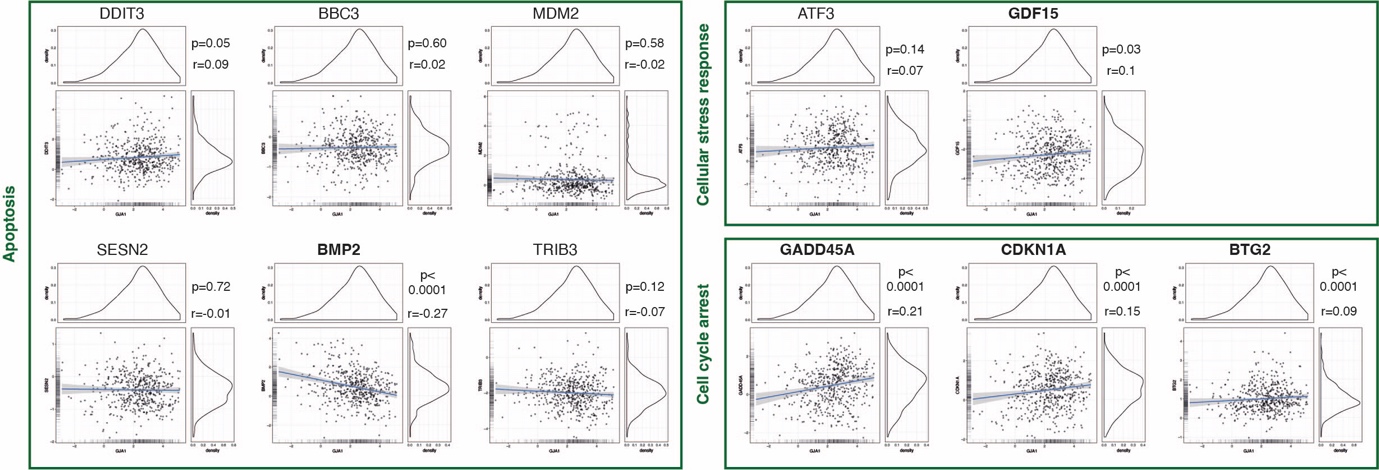


**Supplementary Figure S6: Correlation between *GJA1* expression levels from Agilent-4502A dataset of the TCGA database and genes upregulated under combined TMZ and TO therapy.**

Scatter plots display the genes with their names written as headings above each graph. Genes shown in bold indicate a significant correlation with *GJA1*. Pearson correlation methods were used to determine p- and correlation coefficient (r-values).

Abbreviations: *GJA1*, gene encoding for Cx43; TCGA, The Cancer Gene Atlas; TO, Tonabersat; TMZ, Temozolomide.

**Supplementary Table S1**

| *gRNA name* | *Sequence (5‘-3‘)* |
| --- | --- |
| Cx43-gRNA-TS3 Top | CTTGTCAAGGAGTTTGCCTAgtttt |
| Cx43-gRNA-TS3 Bottom | TAGGCAAACTCCTTGACAAGcggtg |
| Cx43-gRNA-TS4 Top | AAGCCTACTCAACTGCTGGAgtttt |
| Cx43-gRNA-TS4 Bottom | TCCAGCAGTTGAGTAGGCTTcggtg |

Guide RNAs used for gene editing of the *GJA1* gene encoding for Cx43 for G35 cell population.

Abbreviations: Cx43, connexin 43; gRNA, guide RNA.

**Supplementary Table S2**

| **Guide RNA** | **Strand of**  **sgRNA** | **Orientation** | **sgRNA sequence** | **Target Cut Length** | **Target Total Length** |
| --- | --- | --- | --- | --- | --- |
| Cx43/3 | - | antisense | TGAGCCAGGTACAAGAGTGT | 267 | 1149 |
| Non-targeting |  |  | GGTCACCGATCGAGAGCTAG |  |  |

Single guide RNA used for gene editing of the *GJA1*/Cx43 gene for G71 cell population.

Abbreviations: Cx43, connexin 43; sgRNA, single guide RNA.
